# A Systematic Review of Technologies to Teach Control Structures in Preschool Education

**DOI:** 10.3389/fpsyg.2022.911057

**Published:** 2022-09-16

**Authors:** Ewelina Bakala, Anaclara Gerosa, Juan Pablo Hourcade, Gonzalo Tejera, Kerry Peterman, Guillermo Trinidad

**Affiliations:** ^1^Facultad de Ingeniería, Instituto de Computación, Universidad de la República, Montevideo, Uruguay; ^2^Centro Interdisciplinario en Cognición para la Enseñanza y el Aprendizaje, Universidad de la República, Montevideo, Uruguay; ^3^Department of Computer Science, The University of Iowa, Iowa City, IA, United States

**Keywords:** control structures, young children, computational thinking, technology, systematic literature review, preschoolers

## Abstract

There is growing interest in teaching computational thinking (CT) to preschool children given evidence that they are able to understand and use CT concepts. One of the concepts that is central in CT definitions, is the concept of control structures, but it is not clear which tools and activities are successful in teaching it to young learners. This work aims at (1) providing a comprehensive overview of tools that enable preschool children to build programs that include control structures, and (2) analyzing empirical evidence of the usage of these tools to teach control structures to children between 3 and 6. It consists of three parts: systematic literature review (SLR) to identify tools to teach CT to young children, analysis of tools characteristics and the possibilities that they offer to express control structures, and SLR to identify empirical evidence of successful teaching of control structures to young children using relevant tools. This work provides an understanding of the current state of the art and identifies areas that require future exploration.

## 1. Introduction

In 2006, Jeanette Wing popularized the term “Computational thinking” as a universal set of skills which could allow everyone to use computer science concepts for problem solving (Wing, 2006, 2011). Grover (2018) defined two viewpoints on CT: one is that CT is the cognitive or “thinking” counterpart to practicing computer science in CS classrooms. This means CT is a specific characteristic of practicing computer science and is bound to this discipline. The other viewpoint is that CT is a skill to be integrated by other disciplines and it is a way to approach sense-making in different subjects. Wing's original definition of CT was broad enough that it ignited educators and policy-makers' interest in CT (Bocconi et al., 2016). Thus, over the past decade there has been an increase in research around CT interventions targeted at most levels of formal education (Grover and Pea, 2013; Hsu et al., 2018; Yadav et al., 2018; Lyon and Magana, 2020; Stamatios, 2022), its inclusion within other disciplines (Orton et al., 2016; Weintrop et al., 2016; Hickmott et al., 2018), its association with other well-established cognitive skills (Román-González et al., 2017; Robertson et al., 2020; Gerosa et al., 2021; Tsarava et al., 2022), and focusing on creating reliable and valid assessment methods (Tang et al., 2020), amongst others. Moreover, both public and privately-led initiatives have been successfully implemented to foster CT in children and adolescents (Brackmann et al., 2016; Williamson, 2016), as it is regarded as a valuable twenty-first century skill (Yadav et al., 2016).

Several of the most widely accepted and cited definitions of CT propose the use and understanding of control structures such as loops and conditionals as an integral part of CT. For example, Brennan and Resnick (2012) named loops, conditionals and events as central computational concepts in their framework; Grover and Pea (2013) highlighted the use of conditional logic and iteration as well as Shute et al. (2017). In some cases there is no direct reference to control structures in CT definitions, but algorithm design (Khoo, 2020; Saxena et al., 2020) is considered as an essential part of CT. Control structures are basic building components for algorithms (Perkovic, 2015), and therefore an integral part of CT. Moreover, several of the assessments created for evaluating students' CT in formal education include the evaluation of loops and conditionals, such as Román-González (2015) and collaborators' CTt; Relkin et al.'s (2020) TechCheck or the CT sections that were incorporated to the PISA mathematics testing in OECD (2019).

Authors such as Bers (2019, 2020) have argued for the inclusion of CT skills in early childhood education, particularly through the use of robots as an embodied, tangible tool which would be intuitive and developmentally appropriate for young children. Teaching young children CT related concepts prepares them to solve real-life challenges in a logical and systematic way, and some authors consider CT as relevant as reading, writing and mathematics (Sanford and Naidu, 2016). The early exposure to computing has potential to engage both boys and girls mitigating gender-related barriers (Manches and Plowman, 2017; Martin et al., 2017).

**Figure 1:**
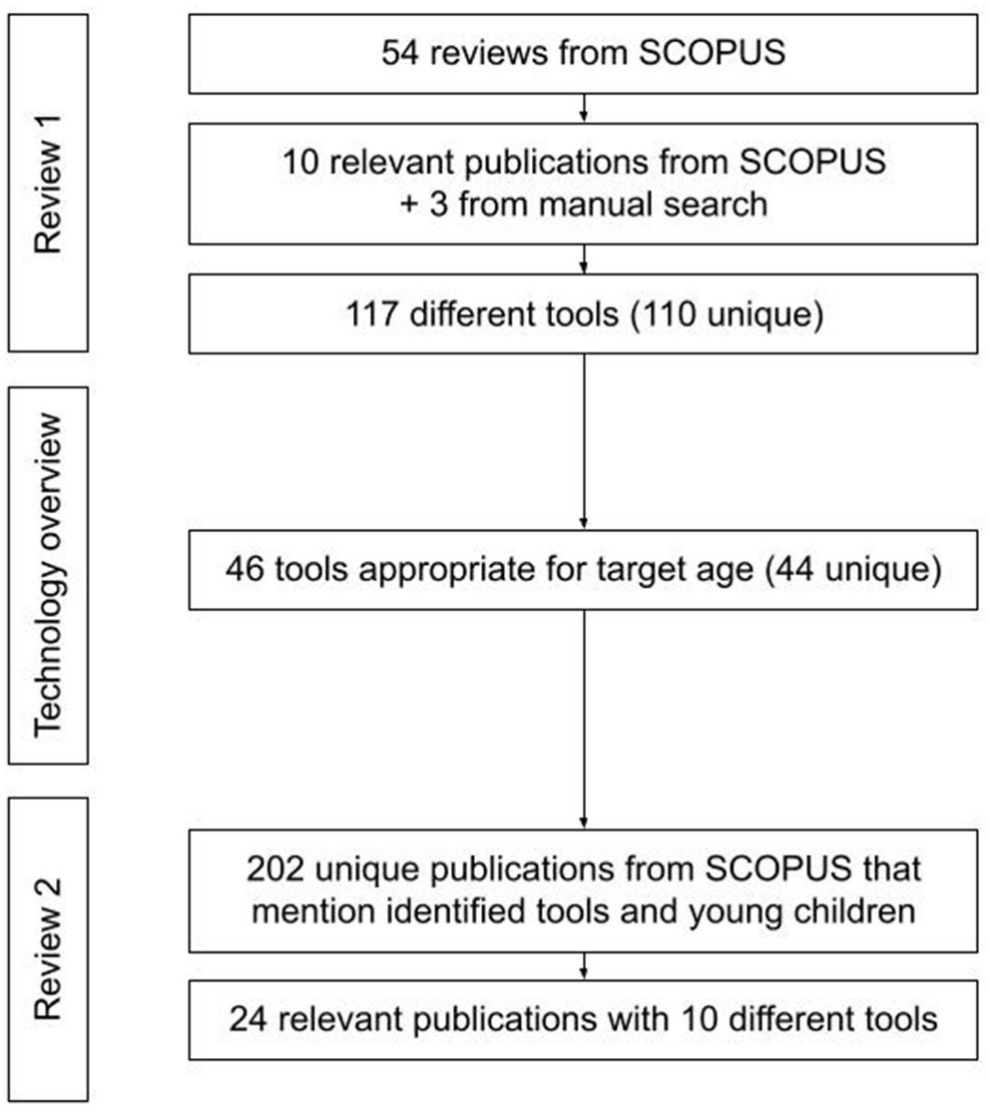
An overview of the research pipeline.

This work aims at presenting the current state of the art of teaching control structures to preliterate children between 3 and 6 years of age using electronic tools (physical, virtual and hybrid systems) that allow users to construct explicit programs. Our work consists of three parts (see Figure 1): (1) review 1: a systematic literature review (SLR) of reviews aimed at identifying technology used to promote CT in young children; (2) technology overview: an analysis of the characteristics of these tools based on information we found in tool websites and user manuals; (3) review 2: a SLR of empirical evidence related to the use of the tools in teaching control structures to preliterate children between the ages of 3 and 6.

The research questions that guide this study are the following:

- What electronic tools exist to support the development of CT in young children? (review 1)
- Which tools are appropriate for preliterate children between the ages of 3 and 6? (technology overview)
- How can children introduce control structures into their programs using electronic tools? (technology overview)
- What tools have been reported to be successful for teaching control structures to young children? (review 2)

In the remainder of the paper we present related works that systematize the knowledge about existing tools that support the development of CT, next we present the methodology and findings of the first SLR that aims to identify existing tools for teaching CT to young children (see Figure 1). In the following step we analyze the tools to identify those that are electronic-based and appropriate for preliterate children between 3 and 6 years old, and provide details related to their price and possibilities that they offer to introduce control structures in children's code. The resulting list of appropriate tools is used in the second SLR to search for empirical evidence related to teaching control structures to young children. The limitations and results are discussed in the final section of the article and conclusions are laid down.

### 1.1. Related Work

Previous work has focused on reviewing technological and unplugged tools to promote CT in young children. However, most of the available reviews on this topic focus on the broad aspects of CT and robotics without specifically analyzing the affordances of particular technological tools for learning a specific concept, such as control structures. For example, Silva et al. (2021) focused on describing the available technology for 2–8 year old children as well as curricula implemented for these ages, while Kakavas and Ugolini (2019) focused on they way the teaching of CT has evolved in primary education in the last decades and was successful in identifying the context in which the technology was implemented and in which way CT was assessed. In a recent review (Bakala et al., 2021) we also analyzed the characteristics of robots and activities used in preschool education to promote CT skills with a focus on empirical research, however the specific ways in which each concept encompassed by CT was targeted was not part of our scope. Recent work by Taslibeyaz et al. (2020) shed light into the way studies with young children considered the concept of CT by analyzing its definitions, which skills were targeted and which variables were assessed and included the technological tools used to promote these skills. However, the implications as to how a specific technology causes this improvement and what are the nuances of using different technological tools were not discussed. Similarly, a recent review by Toh et al. (2016) on the use of robots for young children provided context on the type of study conducted and on the effects of robotics on children's cognitive outcomes as well as parents', educators' and children's opinions regarding the use of these tools. However, the possible benefits are discussed generally regarding robotics and this work does not focus on the outcomes of specific tools. Yu and Roque (2019) provide a comprehensive review of computational toys and kits for young children (7 and under) describing their design features, which computational concepts and practices they target and how they relate to other domains in knowledge. In particular, they analyzed the way conditionals were presented in the technological tools and argued that most of the time conditionals were implemented in an implicit way (thus not represented using explicit if-then statements). In addition, the authors explored the presentation of loops, pointing out many of the available tools used repeat blocks which encapsulated a given sequence, whether digital or concrete. In order to expand upon these findings, this review will focus specifically on the ways technology has implemented control structures and provide an overview of the evidence surrounding these implementations with young children. In this sense, our review will provide a summary of the empirical experiences in which these control structures have been taught to young children and analyze these results. To our knowledge, there isn't thus far a systematic review of literature which focuses on the implementation of control structures and provides a thorough analysis of how technological tools aimed at early childhood allow its users to learn them. In addition, we conducted a SLR on the existing empirical evidence in which control structures have been taught to children, shedding light into which practices and tools are supported by evidence and thus favorable for its inclusion in the classroom.

## 2. SLR of Existing Tools (Review 1)

We used a systematic literature review (Kitchenham et al., 2015) to answer our first research question: What tools exist to support the development of CT in young children?

### 2.1. Methodology

Systematic literature review (SLR) is a method that allows identifying relevant material to a given topic using an objective, analytical, and repeatable approach (Kitchenham et al., 2015). We carried out our literature review following the PRISMA guidelines (Moher et al., 2009). Four reviewers participated in the review process. Firstly, they defined the search term, inclusion and exclusion criteria, and data to extract from the publications. Secondly, two reviewers analyzed the publications to identify the relevant articles. One reviewer extracted the tools from relevant articles. A quality assessment stage was not included, as we were not interested in filtering out low quality studies since we still reviewed each tool or investigating changes in quality over time.

#### 2.1.1. Search Strategy

To identify reviews of technology to support the development of CT in young children we applied an automated search (Kitchenham et al., 2015) in the Scopus search engine (Elsevier Scopus, 2022). The search term was the following:

TITLE-ABS-KEY ( ( ( review AND {computational thinking} AND ( preschool OR child OR {early age} OR kindergarten OR {lower education} OR {early years} OR {elementary education} OR {young learners} OR {primary school} OR {primary education} OR k-6 OR k-8 OR childhood ) ) ) )

We used three keywords: review, computational thinking and childhood (and synonyms) to search in the title, abstract, and keywords.

#### 2.1.2. Study Selection

We defined the following inclusion criteria for the studies' selection:

- Articles that review electronic-based tools to promote the development of CT in young children.
- Publications focused on children between 3 and 5 years old, including 6 years old, if attending pre-primary school educational level.

Exclusion criteria were:

- Articles written in a language other than English or Spanish.
- Publications that target children older than 6 years.
- Articles limited to unplugged tools.
- Case studies.
- Conference proceedings.

The first round of the selection was made based on the information available in the abstract. Two researchers applied the criteria independently and filter out publications that do not review tools focused on promoting CT in young children. The articles were tagged as “relevant” or “irrelevant.” If an article was classified differently by the reviewers, the full text was reviewed. If there were doubts about an article, they were discussed with two other reviewers that supervised this revision step. Also the articles that were considered relevant by both reviewers were analyzed in detail to confirm or reject their relevance.

#### 2.1.3. Data Extraction

We used a spreadsheet to extract tools found in the publications and articles that mention each tool. We sorted each tool using categories that we developed (see Section 2.4).

### 2.2. Findings

### 2.3. Relevant Articles

**Figure 2:**
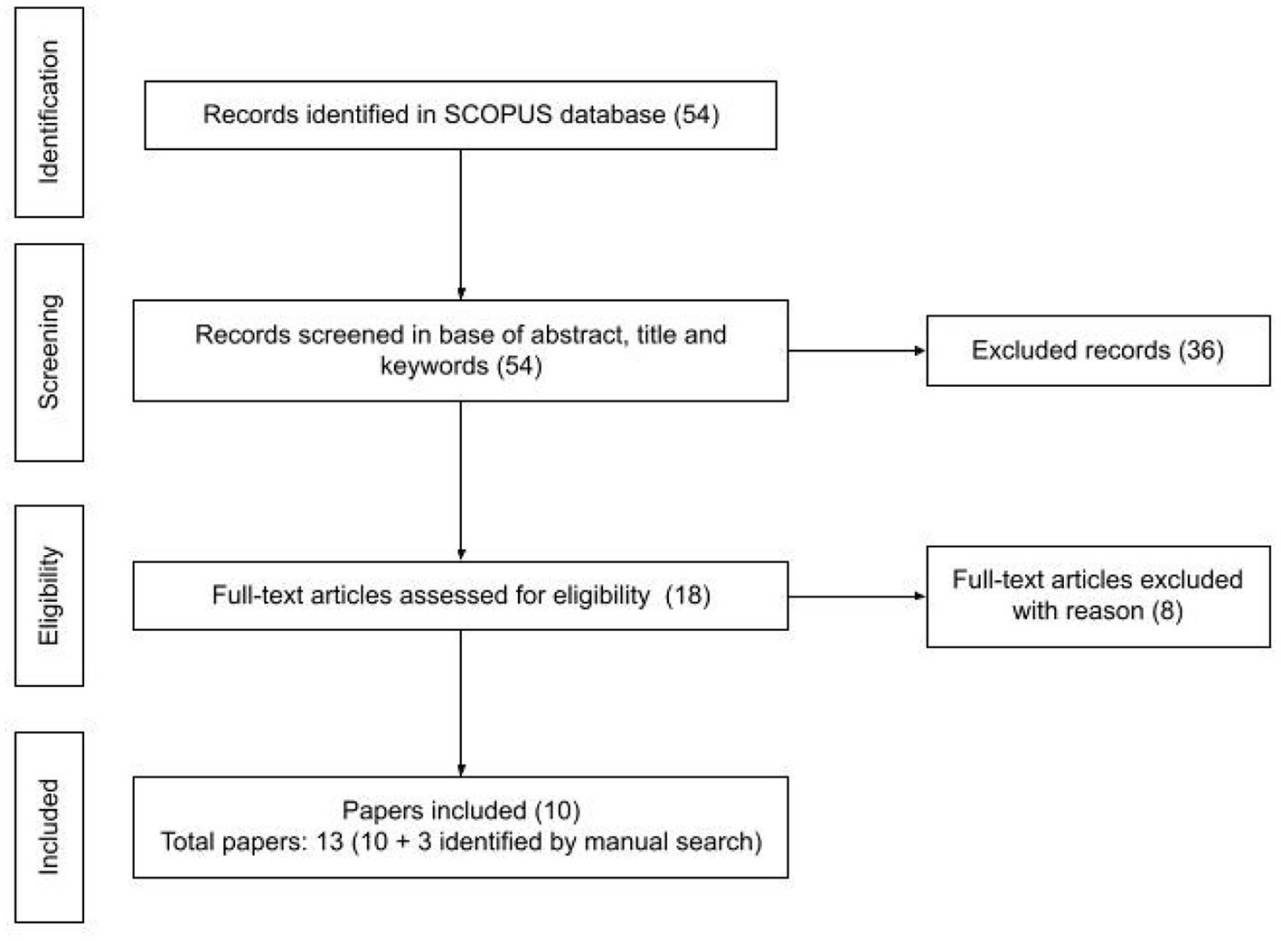
Steps of the selection process of the first SLR. Reported in line with the PRISMA statement (Moher et al., 2009).

The search was conducted on 6th of August 2021 and we obtained 54 articles to review (see Figure 2). In the screening phase the reviewers tagged identically 51 of 54 articles reaching an agreement of 0.94%. In the selection process we identified 10 articles relevant for this study. We added to our analysis 3 articles (Kakavas and Ugolini, 2019; Papadakis, 2020; Silva et al., 2021) that were identified by the manual search and that we considered a valuable source of information-Kakavas and Ugolini (2019) that was not indexed by Scopus, Papadakis (2020) that does not contain the word “review” in title, abstract and keywords and Silva et al. (2021) that is a preprint submitted to Elsevier.

A total of 13 articles (see Table 1) were used to elaborate the list of relevant tools. All the articles were published between 2018 and 2021.

**Table 1:** 13 relevant publications that we identified in the first SLR.

**References**	**Title**
Bakala et al. (2021)	Preschool children, robots, and computational thinking: A systematic review
Papadakis (2021)	The Impact of Coding Apps to Support Young Children in Computational Thinking and Computational Fluency. A Literature Review
Fagerlund et al. (2021)	Computational thinking in programming with Scratch in primary schools: A systematic review
Yang et al. (2020)	The influence of robots on students' computational thinking: A literature review
Pedersen et al. (2020)	The effect of commercially available educational robotics: A systematic review
Umam et al. (2019)	Literature review of robotics learning devices to facilitate the development of computational thinking in early childhood
Isnaini et al. (2019)	Robotics-based learning to support computational thinking skills in early childhood
Yu and Roque (2019)	A review of computational toys and kits for young children
Ching et al. (2018)	Developing Computational Thinking with Educational Technologies for Young Learners
Ioannou and Makridou (2018)	Exploring the potentials of educational robotics in the development of computational thinking: A summary of current research and practical proposal for future work
Silva et al. (2021)	A Systematic Review of Computational Thinking in Early Ages
Papadakis (2020)	Robots and Robotics Kits for Early Childhood and First School Age
Kakavas and Ugolini (2019)	Computational thinking in primary education: a systematic literature review

### 2.4. Categories to Classify the Tools

**Figure 3:**
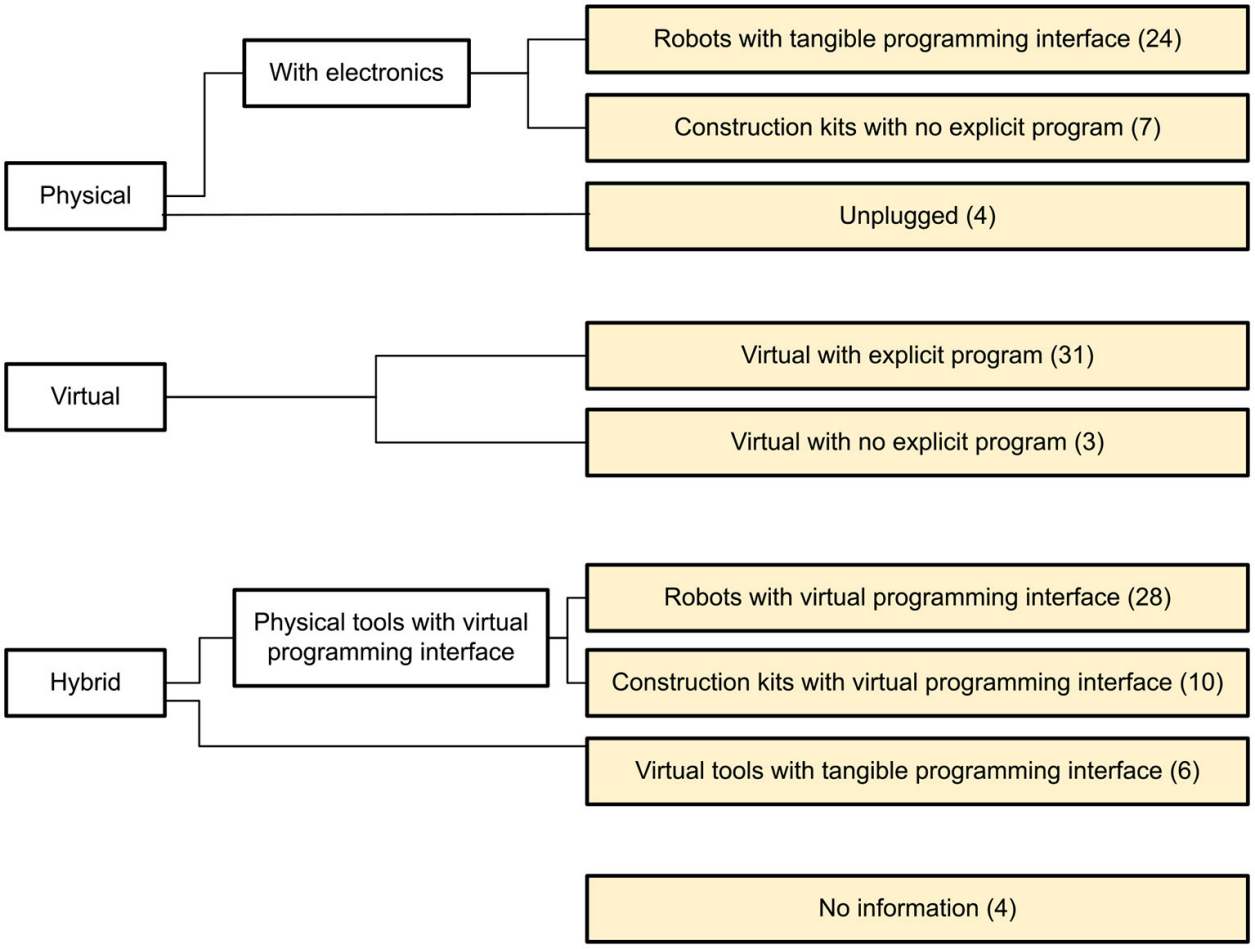
Categories developed to classify the physical aspect of the tools.

To classify the tools we adapted and expanded categories proposed by Yu and Roque (2019). We obtained 4 main categories (see Figure 3): Physical, Virtual, Hybrid and No information. We divided Physical, Virtual and Hybrid into sub-categories and obtained 9 categories which we used to classify existing tools: Robots with tangible programming interface, Construction kits with no explicit program, Unplugged, Virtual with explicit program, Virtual with no explicit program, Robots with virtual programming interface, Construction kits with virtual programming interface, Virtual tools with tangible programming interface, No information. In the Figure 3 there are more than 8 categories, but only those highlighted in yellow were used to classify the tools.

We used the category Physical for tools that are fully tangible and do not require screen-based applications. We divided it into Unplugged and Physical tools with electronics. The last category was composed of Robots with tangible programming interface and Construction kits with no explicit program. The category Construction kits with no explicit program contains electronic building blocks that can be connected together to cause certain behavior of the system, but do not require the user to write an explicit program.

Virtual contains fully screen-based tools, such as desktop, mobile, or web apps. This category encompasses tools that do not require the user to write an explicit program (e.g., tools like CompThink App where the user has to solve logical problems without writing code) and those which need an explicit program.

Hybrid tools combine physical and virtual parts. We divided them into Virtual tools with tangible programming interface or Physical tools with virtual programming interface. The first category consists of applications with tangible programming interfaces. The second category is composed of Robots with virtual programming interface and Construction kits with virtual programming interface. The last category contains embedded systems like Arduino that can be programmed using a virtual programming interface.

The “No information” category was used if there was no information about the tool that could be used to classify it.

### 2.5. Tools

From the 13 relevant publications we extracted 110 tools (106 unique tools). In the case of Code & Go Robot Mouse, we found three different names that referred to this tool: Robot Mouse (Yu and Roque, 2019; Pedersen et al., 2020), Colby robotic mouse (Papadakis, 2020; Bakala et al., 2021) and Code & Go Robot Mouse (Ching et al., 2018; Silva et al., 2021), and we analyzed it as one single tool.

While reviewing the tools mentioned in the articles we found in external sources 4 more tools that we considered relevant for our work. We added Qobo (Physical and Hybrid), VEX 123 (Physical and Hybrid), Sphero indi (Physical and Hybrid), Scottie Go (Virtual) and ended up with a total of 117 tools (110 unique tools, see Table 2).

**Table 2:** 117 tools extracted from 13 relevant publications that we identified in the first SLR.

**Tool type**	**Name**	**Target age**	**Exclusion reason [Age, RRS (require reading skills), Unplugged, No info, No program]**	**Source**
Robots with tangible programming interface	Bee Bot	3+		Umam et al. (2019), Yu and Roque (2019), Papadakis (2020), Pedersen et al. (2020), Silva et al. (2021), Bakala et al. (2021), Yang et al. (2020)
	Blue Bot	3–11		Yu and Roque (2019), Papadakis (2020), Pedersen et al. (2020), Silva et al. (2021)
	Botley	5+		Papadakis (2020)
	Code-a-Pillar	3–6		Ching et al. (2018), Yu and Roque (2019), Papadakis (2020)
	Cubetto	3–9		Isnaini et al. (2019), Ching et al. (2018), Yu and Roque (2019), Papadakis (2020), Umam et al. (2019), Pedersen et al. (2020)
	Dr. Wagon	6–12	RRS	Yu and Roque (2019)
	Edison robot	4–16	No program	Papadakis (2020), Pedersen et al. (2020)
	KIBO	4–7		Ching et al. (2018), Umam et al. (2019), Yu and Roque (2019), Papadakis (2020), Pedersen et al. (2020), Silva et al. (2021), Bakala et al. (2021), Yang et al. (2020);
	KIWI	5–7		Bakala et al. (2021)
	KUBO robot	4–10		Papadakis (2020), Pedersen et al. (2020)
	Matatalab Coding Set	4–9		Papadakis (2020)
	mTiny	4+		Papadakis (2020)
	Ozobot Evo	5–18		Papadakis (2020)
	Ozobot Bit	6+		Papadakis (2020), Bakala et al. (2021)
	Plobot	4+		Yu and Roque (2019)
	Pro-bot	3+		Yu and Roque (2019), Papadakis (2020), Pedersen et al. (2020), Silva et al. (2021)
	Qobo	3–8		Manual
	Roamer	4–13	No program	Papadakis (2020)
	Robot Mind Designer	7+	Age	Papadakis (2020)
	Code and Go Robot Mouse	4–9		Ching et al. (2018), Yu and Roque (2019), Papadakis (2020), Pedersen et al. (2020), Silva et al. (2021), Bakala et al. (2021);
	Robotito	4–6		Silva et al. (2021)
	Sphero indi	4–8		Manual
	TurtleBot	No info		Bakala et al. (2021)
	VEX 123	4–9		Manual
Construction kits with no explicit program	Cubelets	4+	No program	Papadakis (2020), Pedersen et al. (2020)
	Curlybot	No info	No program	Yu and Roque (2019)
	Electronic Blocks	4–6	No program	Yu and Roque (2019)
	LittleBits	8+	No program	Kakavas and Ugolini (2019), Pedersen et al. (2020)
	Makeblock Neuron	6+	No program	Pedersen et al. (2020)
	roBlocks	9+	No program	Yu and Roque (2019)
	Romibo	No info	No program	Pedersen et al. (2020)
Unplugged	Code Monkey Island	6+	Unplugged	Ching et al. (2018)
	Happy Maps	No info	Unplugged	Silva et al. (2021)
	Hello Ruby	5+	Unplugged	Yu and Roque (2019)
	Robot Turtles	4+	Unplugged	Ching et al. (2018), Yu and Roque (2019)
Virtual with explicit program	AgentCubes	8+	Age	Kakavas and Ugolini (2019)
	AgentSheets	11–13	Age	Kakavas and Ugolini (2019)
	Alice	11+	Age	Kakavas and Ugolini (2019)
	BOTS	5–18		Kakavas and Ugolini (2019)
	Cargo-Bot	10–18	Age	Ching et al. (2018), Yu and Roque (2019)
	Codeable Crafts	4+		Yu and Roque (2019)
	Code.org	4+		Ching et al. (2018), Silva et al. (2021)
	CodyColor	0+	No program	Silva et al. (2021)
	CTSiM	5–18	RRS	Kakavas and Ugolini (2019)
	Daisy the Dinosaur	7+	Age	Papadakis (2021)
	FormulaT Racing	7–13	Age	Kakavas and Ugolini (2019)
	Hopescotch	10–16	Age	Ching et al. (2018)
	Kodable	4–10		Ching et al. (2018), Papadakis (2021), Silva et al. (2021)
	Kodetu	9–17	Age	Kakavas and Ugolini (2019)
	Kodu	9+	Age	Kakavas and Ugolini (2019)
	Legato	4–11	No program	Ching et al. (2018), Silva et al. (2021)
	LightBot	9+	Age	Ching et al. (2018), Yu and Roque (2019), Kakavas and Ugolini (2019), Papadakis (2021), Silva et al. (2021)
	LightBotJr	4–8		Ching et al. (2018), Silva et al. (2021)
	MiniColon game	8–9	Age	Kakavas and Ugolini (2019)
	Move the turtle	5+		Yu and Roque (2019)
	RoboZZle	6–7		Yu and Roque (2019)
	Run Marco!	4+		Yu and Roque (2019)
	Scratch	8–16	Age	Ching et al. (2018), Isnaini et al. (2019), Kakavas and Ugolini (2019), Fagerlund et al. (2021)
	ScratchJr	5–7		Kakavas and Ugolini (2019), Yu and Roque (2019), Ching et al. (2018), Papadakis (2021), Silva et al. (2021)
	Story-Writing-Coding engine	5–11	RRS	Kakavas and Ugolini (2019)
	The Foos	5+		Yu and Roque (2019), Silva et al. (2021)
	Tuk Tuk (standard)	5–14	RRS	Silva et al. (2021)
	Tynker: Coding for Kids	5–14		Ching et al. (2018)
	VBOT	14+	Age	Ioannou and Makridou (2018), Yang et al. (2020)
	ViMAP	8–10	Age	Kakavas and Ugolini (2019)
	Zoombinis game	8+	Age	Kakavas and Ugolini (2019)
Virtual with no explicit program	CompThink App	5–11	No program	Kakavas and Ugolini (2019)
	PhysGramming	6–7	No program	Silva et al. (2021)
	Tuk Tuk (junior)	5–6	No prgram	Silva et al. (2021)
Robots with virtual programming interface	Blue Bot	3–11		Yu and Roque (2019), Papadakis (2020), Pedersen et al. (2020), Silva et al. (2021)
	CHERP	5–6		Ioannou and Makridou (2018), Kakavas and Ugolini (2019)
	Codey Rocky	5–11	RRS	Pedersen et al. (2020)
	COJI	6+		Yu and Roque (2019), Papadakis (2020)
	Cozmo	8–11	Age	Pedersen et al. (2020)
	Dash and/or Dot	6+		Ching et al. (2018), Yu and Roque (2019), Papadakis (2020), Pedersen et al. (2020)
	Finch	5+		Papadakis (2020)
	LEGO Boost	7–12	Age	Pedersen et al. (2020)
	LEGO Education WeDo	7+	Age	Kakavas and Ugolini (2019), Isnaini et al. (2019), Ching et al. (2018), Papadakis (2020), Silva et al. (2021), Pedersen et al. (2020), Umam et al. (2019), Bakala et al. (2021)
	LEGO Mindstorm	10+	Age	Kakavas and Ugolini (2019), Ching et al. (2018), Ioannou and Makridou (2018), Pedersen et al. (2020), Bakala et al. (2021)
	Max Tobo coding robot	6+	RRS	Papadakis (2020)
	mBot	8+	Age	Pedersen et al. (2020), Silva et al. (2021)
	MeeperBots	5–12	RRS	Yu and Roque (2019), Papadakis (2020)
	Mind designer robot	7+	Age	Papadakis (2020)
	MiP	8–15	Age	Pedersen et al. (2020)
	MU Spacebot	8+	Age	Pedersen et al. (2020)
	NAO	5–18	RRS	Kakavas and Ugolini (2019), Pedersen et al. (2020)
	Qobo	3–8	RRS	Manual
	ROBOTC Graphical	No info	RRS	Kakavas and Ugolini (2019)
	Scribbler	14+	Age	Pedersen et al. (2020)
	Sphero Ollie	8–14	Age	Pedersen et al. (2020)
	Sphero indi	4–8		Manual
	Sphero mini	8+	Age	Papadakis (2020)
	The Coffee Platform	No info	RRS	Ioannou and Makridou (2018)
	Thymio	6+		Yu and Roque (2019), Papadakis (2020), Pedersen et al. (2020)
	Tinkerbots	5+		Papadakis (2020)
	VEX 123	4–9	RRS	Manual
	VBOT	11–18	Age	Ioannou and Makridou (2018), Yang et al. (2020)
Construction kits with virtual programming interface	An ultra-low cost line follower Robotic	16–18	Age	Yang et al. (2020)
	Arduino+scratch	7–13	Age	Yang et al. (2020)
	CyberPLAYce	8–12	Age	Kakavas and Ugolini (2019)
	GoGo Board	10–18	Age	Ioannou and Makridou (2018)
	Hummingbird Robotics Kit	9–18	RRS	Pedersen et al. (2020)
	Makeblock Neuron	6+		Pedersen et al. (2020)
	micro:bit	8–14	Age	Pedersen et al. (2020)
	Scratch 4 Arduino, S4A)	8–17	Age	Kakavas and Ugolini (2019)
	ultimate	12+	Age	Pedersen et al. (2020)
	VEX IQ	11+	Age	Pedersen et al. (2020)
Virtual tools with tangible programming interface	Puzzlets Starter Pack	6+		Yu and Roque (2019)
	Roberto	4+		Yu and Roque (2019)
	Scottie Go	4–15		Manual
	Coding Awbie	5–11		Ching et al. (2018), Papadakis (2020), Silva et al. (2021), Yu and Roque (2019)
	Tabletop puzzle block system	4–5		Yu and Roque (2019)
	T-Maze	5–9		Kakavas and Ugolini (2019), Silva et al. (2021)
No info	LEGO	No info	No info	Yang et al. (2020), Bakala et al. (2021)
	Ozobot	No info	No info	Kakavas and Ugolini (2019), Pedersen et al. (2020)
	Robo Cup Junior	no info	No info	Isnaini et al. (2019)
	Robotis and roboplus software	No info	No info	Ioannou and Makridou (2018)

We classified 35 as Physical, 34 as Virtual, 44 as Hybrid and 4 as No information (see Figure 3).

It is important to say that seven tools were present in more than one category (Blue-Bot, Qobo, VEX 123, Sphero indi, VBOT, Makeblock Neuron, Tuk Tuk). For example, Blue-Bot is a robot that can be programmed using buttons on its back and because of that it belongs to the category Robots with tangible programming interface, but there is also a possibility to program it using an application, so it was also classified as a Robot with a virtual programming interface. That is why we refer to 110 unique tools, although we analyzed 117 relevant tools that included duplicated items.

In three cases (Ozobot, LEGO, Robotis and roboplus software) the names that we found in publications were names of brands, not names of specific tools, so it was impossible to classify them, and they were categorized as No information. One publication mentioned Robo Cup Junior as a tool. As far as we know RoboCup Junior (RoboCupJunior, 2022) is an educational initiative, not one particular technology, so we categorized this item as No information as well.

## 3. Technology Overview

The first aim of this part of our study was to identify how young, preliterate children can introduce conditionals and iterations into their programs using existing tools. This section is motivated by the following research questions:

- Which tools are appropriate for preliterate children between the ages of 3 and 6?
- How can children introduce control structures into their programs using electronic tools?

### 3.1. Methodology

Four reviewers participated in the revision of existing tools. Two of them reviewed the available online information and extracted the information of interest. The other two participated in the definition of the categories to classify tools' characteristics and helped to classify doubtful cases.

#### 3.1.1. Tools Selection

We were interested in electronic tools that allow users to construct explicit programs, so we did not further analyze the tools classified as Unplugged, Construction kits with no explicit program, Virtual with no explicit program, and No information.

We identified the relevant tools by filtering out those not appropriate for children between 3 and 6 - tools that target children older than 6 years old or that should be programmed using interfaces that require reading skills (see Table 2). During tool selection we first analyzed the target age of each tool. If the information of the target age was expressed using educational levels like “elementary school” or “kindergarten” we translated this information into age using the United States educational system as reference. If the tool was designed for children older than 6, we tagged it as inappropriate and did not analyze its programming interfaces. If the age was of our interest, we proceeded with the inspection of the user interface. In many cases hybrid tools offered different programming languages/interfaces to cover a wide age spectrum of users, for example, Finch Robot can be programmed using 8 different programming languages and its promotional video states that it is suitable for users from “from kindergarten to college.” In those cases we evaluated only programming languages appropriate for preliterate children. If there was no interface suitable for preschoolers, we marked it as a tool that requires reading skills.

#### 3.1.2. Data Extraction

To collect the information about the tools we reviewed the official websites, video material provided by the manufacturer, online manuals, as well as, youtube videos and amazon websites.

During data extraction we were interested in classifying different types of control structures that can be used with each tool, so we defined categories that we present in Sections 3.2.2.1, 3.2.2.2.

### 3.2. Findings

#### 3.2.1. Tools Selection

We identified 46 tools (44 unique) appropriate for preliterate children (see Table 3). Twenty Robots with tangible programming interface, 11 Virtual with explicit program and 15 Hybrid tools: 8 Robots with virtual programming interface, 1 Construction kit with virtual programming interface and 6 Virtual tools with tangible programming interface. Two tools (Blue Bot and Sphero indi) were classified as both: Robots with tangible programming interface and Robots with virtual programming interface.

**Table 3:** An overview of 46 relevant tools considering their price and possibilities to incorporate control structures into the code.

**Classification**	**Tool name**	**Conditionals [Predefined connection, Free connection, Free condition building]**	**Integration with the main program [Integrated if, Blocking event, Interruption, Parallel execution]**	**Number of repetitions [Fixed number of repetitions, Configurable number of repetitions, Infinite loop]**	**Number of repeated commands [Single command repetition, Multiple command repetition]**	**Price (USD)**
Robots with tangible programming interface	Bee Bot	–	–	–	–	85
	Blue Bot	–	–	–	–	104
	Botley	Free connection	Interruption	Configurable number of repetitions	Multiple command repetition	47
	Code–a–Pillar	–	–	Configurable number of repetitions	Single command repetition	148 (new version) or 35 (old)
	Cubetto	–	–		–	225
	KIBO	Free connection + Free condition building	Blocking event + Integrated if	Configurable number of repetitions + Infinite loop	Multiple command repetition	230 to 610
	KIWI	Free connection + Free condition building	Blocking event + Integrated if	Configurable number of repetitions + Infinite loop	Multiple command repetition	Unavailable
	KUBO robot	–	–	Configurable number of repetitions	Multiple command repetition	310 to 396
	Matatalab Coding Set	Free connection + Free condition building	Blocking event	Configurable number of repetitions	Multiple command repetition	169
	mTiny	–	–	Configurable number of repetitions	Multiple command repetition	120
	Ozobot Evo	Predefined connection	Integrated if	–	–	175
	Ozobot Bit	Predefined connection	Integrated if	–	–	Unavailable
	Plobot	Free connection	Blocking event	–	–	Unavailable
	Pro–bot	Free connection	Interruption	Configurable number of repetitions	Multiple command repetition	150
	Qobo	Predefined connection	Blocking event + Integrated if	Fixed number of repetitions	Multiple command repetition	60
	Robot Mouse	–	–	–	–	60
	Robotito	Predefined connection	Interruption	–	–	Unavailable
	Sphero indi	Predefined connection	Interruption	–	–	100
	TurtleBot	Predefined connection	Integrated if	–	–	105
	VEX 123	Free connection	Integrated if	Fixed number of repetitions + Configurable number of repetitions + Infinite loop	Single command repetition + Multiple command repetition	119
Virtual with explicit program	BOTS	Free condition building	Integrated if	Configurable number of repetitions	Multiple command repetition	Unavailable
	Codeable Crafts	Free connection	Parallel execution	Configurable number of repetitions + Infinite loop	Single command repetition + Multiple command repetition	Free
	Code.org	Free condition building	Interruption	Configurable number of repetitions	Multiple command repetition	Free
	Kodable	Free connection	Interruption	Configurable number of repetitions	Multiple command repetition	Free–2000 yearly
	LightBotJr	–	–	Configurable number of repetitions + Infinite loop	Multiple command repetition	2.99
	Move the turtle	Free condition building	Integrated if	Configurable number of repetitions	Multiple command repetition	3.99
	RoboZZle	Free connection	Interruption	Configurable number of repetitions + Infinite loop	Multiple command repetition	Free
	Run Marco!	Free condition building	Integrated if	Configurable number of repetitions	Multiple command repetition	Free
	ScratchJr	Free connection	Parallel execution	Configurable number of repetitions + Infinite loop	Single command repetition + Multiple command repetition	Free
	The Foos	Free condition building	Integrated if	Configurable number of repetitions + Infinite loop	Multiple command repetition	Free
	Tynker: Coding for Kids	Free connection	Integrated if + Interruption	Configurable number of repetitions	Single command repetition + Multiple command repetition	Free
Robots with virtual programming interface	Blue Bot	–	–	Configurable number of repetitions	Multiple command repetition	104
	CHERP	Free connection + Free condition building	Blocking event + Integrated if	Configurable number of repetitions + Infinite loop	Multiple command repetition	Unavailable
	COJI	Free connection	Interruption	–	–	32
	Dash and/or Dot	Free connection + Free condition building	Blocking event	Infinite loop	Multiple command repetition	150
	Finch	Free connection	Parallel execution	Configurable number of repetitions	Multiple command repetition	139
	Sphero indi	Free connection	Interruption	–	–	100
	Thymio	Free connection	Interruption	–	–	160
	Tinkerbots	–	–	Configurable number of repetitions	Single command repetition + Multiple command repetition	149
Construction kits with virtual programming interface	Makeblock Neuron	Free condition building	Integrated if	–	–	Unavailable
Virtual tools with tangible programming interface	Puzzlets Starter Pack	–	–	Configurable number of repetitions	Single command repetition	147
	Roberto	Free condition building	Blocking event	Infinite loop	Multiple command repetition	Unavailable
	Scottie Go	Free condition building	Integrated if	Configurable number of repetitions + Infinite loop	Single command repetition + Multiple command repetition	45–74
	Coding Awbie	Free connection	Integrated if	Configurable number of repetitions	Single command repetition + Multiple command repetition	99
	Tabletop puzzle block system	–	–	–	–	Unavailable
	T–Maze	Predefined connection	Blocking event	Configurable number of repetitions	Multiple command repetition	Unavailable

There were three tools that we analyzed together: KIBO, KIWI and CHERP. KIBO is a robot currently available in the market, formerly known as “KIWI” or Kids Invent with Imagination (Tufts University, 2022). CHERP is a programming language that is used to program KIBO and KIWI, so evaluating CHERP is equivalent to evaluating KIBO and KIWI.

In the case of some tools, the programming interface contained images which made it accessible for preliterate children, but we had the impression that the systems were designed for children older than our target age. They contained text-based challenges (Scottie Go) and menus (BOTS, Neuron App, Move the turtle, RoboZZle), design that we consider unattractive for young children (RoboZZle, BOTS), text-based options with no associated image (“tap” event in Roberto), or comparisons involving high numeric values (Neuron App). Although these tools raised some doubts, we decided to include them in our analysis as we wanted to provide an inclusive overview of the existing tools.

#### 3.2.2. Categories to Classify Control Structures

We developed categories related to the use of control structures to classify tools suitable for young children (see Table 3) that we identified during tools selection step (see Section 3.2.1).

##### 3.2.2.1. Conditionals

To identify how the children can introduce decision making based on certain conditions into their programs we reviewed the programming interfaces and classified the existing tools with categories that we defined in an iterative process. Introducing conditions in the code was typically based on conditional branches (e.g., if-else structures) or based on events (e.g., blocking the program execution until some event occurs). From now on we will refer to those two forms of incorporation of conditions into the code as “conditionals.”

To classify the degree of liberty that the children have while using and building conditionals in their programs, we propose three levels, ordered by increasing complexity for the user:

1. Predefined connection of condition and action: it is possible to use a predefined programming statement that connects an event with an action. For example, the Qobo robot detects coding cards below it and acts according to the statement stored in the card. It has a specific card for conditional turning - if the robot passes over a card with a banana before passing over a bifurcation card, it turns left, but if it passes over a card with an apple, it turns right. Neither the condition nor the resulting action can be modified by the user.
2. Free connection of predefined condition and predefined action: it is possible to combine predefined conditions with predefined actions to build custom conditionals. For example, the Sphero Edu Jr application (see **Table 5**) allows users to associate a color sensed by the robot (predefined condition) with an action involving movement, light, and/or sound of the Sphero indi robot (predefined actions). The user needs at least two programming statements (condition and action) to build a conditional. In the case of Kodable and RoboZZle these two statements are combined in one coding block: the background color of the block defines the condition (e.g., “if the tile is pink”) and the arrow, the action (e.g., “go right”). The user is able to modify both: the background color and the arrow direction (see **Table 5**).
3. Free condition building: there are blocks that have to be combined with condition and action. In these cases the user has to use at least three components (bridge-block, condition, and action) to define a conditional. For example, the Matatalab Coding Set contains a “wait until” block that should be combined with a condition (e.g., dark or light) and a sequence of actions in order to build conditionals.

We provide the description and graphical example for each tool that supports conditionals in three tables: Table 4 gathers tools that implement the first level, Table 5 corresponding to the second level, and Table 6 corresponding to the last one.

**Table 4:** Tools that allow building conditionals categorized as “Predefined connection of condition and action”.

**Tool**	**Description**	**Reference image**
Qobo	Specific card for conditional turning - if the robot passes over a card with a banana before passing over a bifurcation card, it turns left, but if it passes over a card with an apple, it turns right.	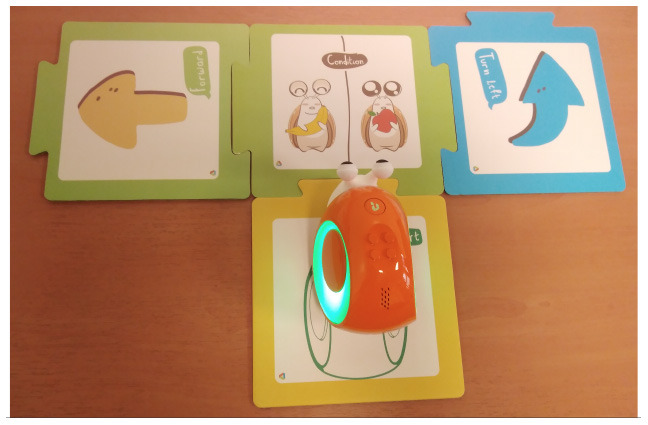
Sphero indi	Color cards that the robot senses in the environment code robots' actions. Image provided by Sphero (2022).	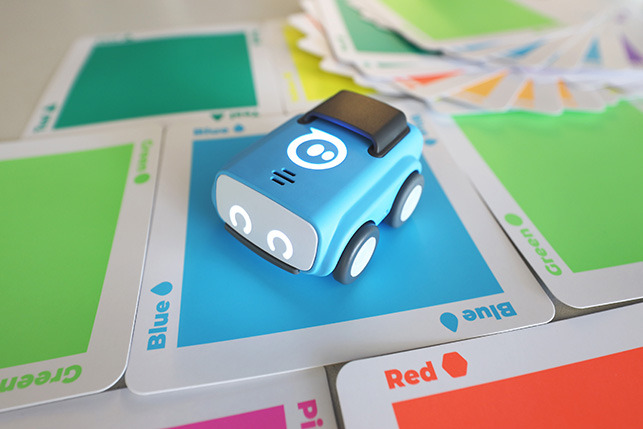
Ozobot Bit and Evo	Color lines that the robot senses in the environment code robots' actions.	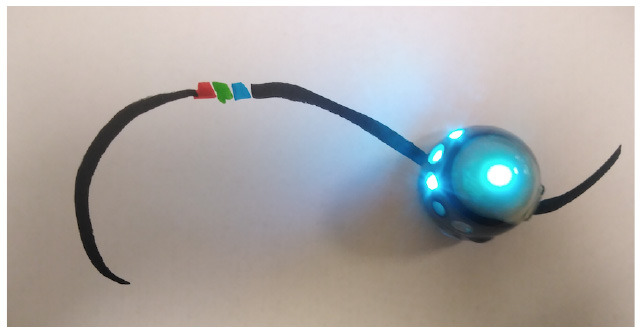
Robotito	Color cards that the robot senses in the environment code robots' actions.	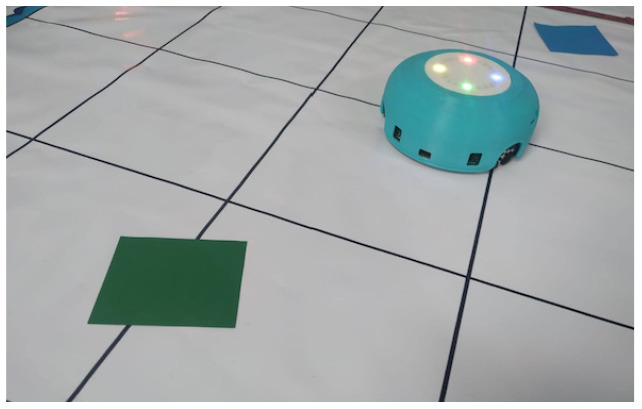
TurtleBot	Color codes that the robot senses in the environment code robots' actions.	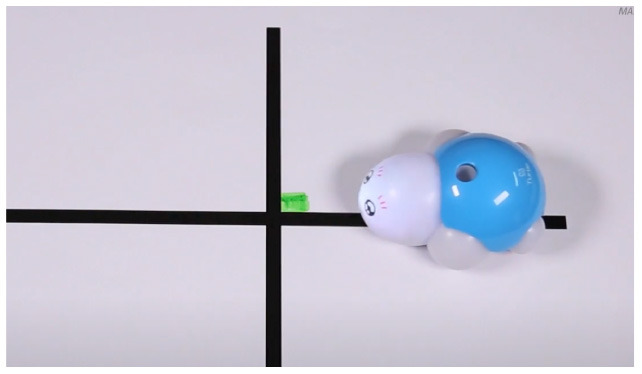

**Table 5:** Tools that allow building conditionals categorized as “Free connection of predefined condition and predefined action”.

**Tool**	**Description**	**Reference image**
KIBO	“Wait for clap” block stops the program execution until the clap is sensed.	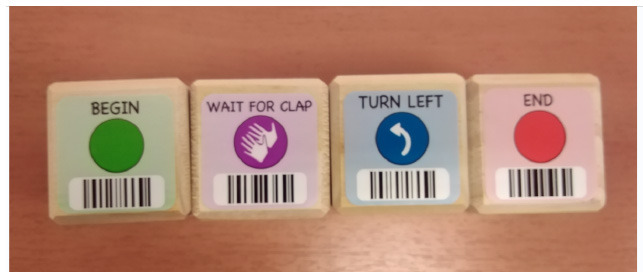
Botley	Botley's control provides an “object detection” button that is used to store the program that is executed when an obstacle is detected in front of the robot.	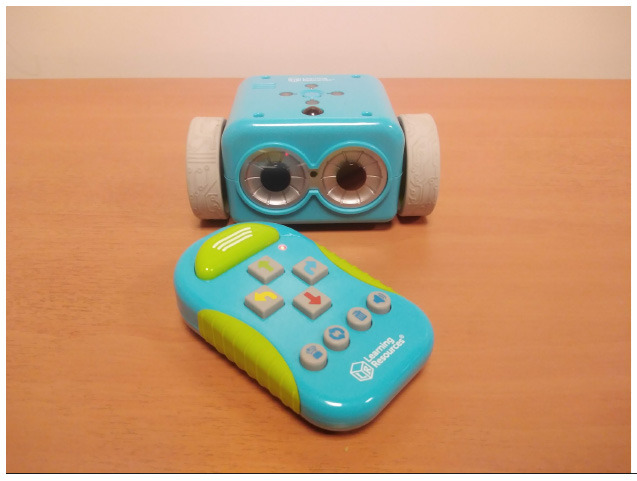
Matatalab Coding Set	Two robots can send messages to each other. “Message received” block is used to define the robot's action when a message is received. The block is available in Matatalab Sensor Add-on (2022).	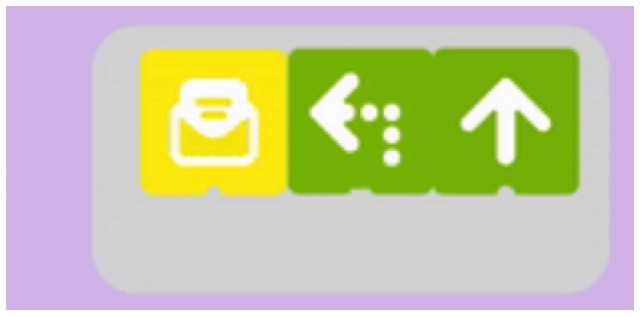
Plobot	“Listen” card blocks the program execution until Plobot detects a sound louder than a soft clap.	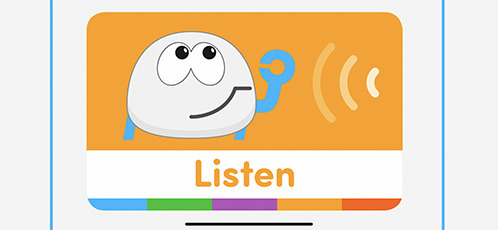
Pro-bot	Procedure numbers 33 to 37 are activated with sensors. For example, the procedure associated with a light sensor runs when the light sensor goes from dark to light.	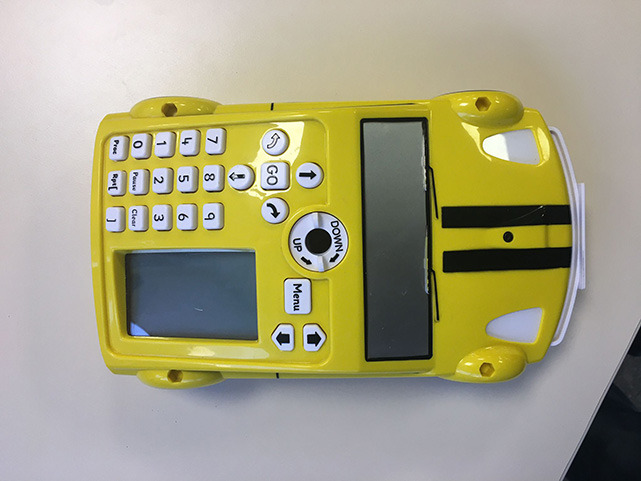
VEX 123	Control cards make use of sensors to check conditions.	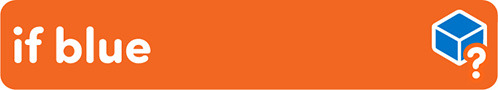
ScratchJr and Codeable Crafts	Events related to characters like “on bump” or “on tap” can be associated with actions.	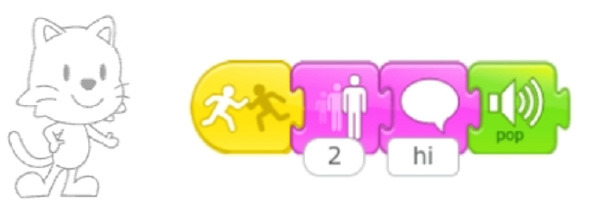
Kodable	The background color of the block defines the condition (e.g., “if the tile is pink”) and the arrow, the action (e.g., “go right”). Image used with permission of Kodable (2022).	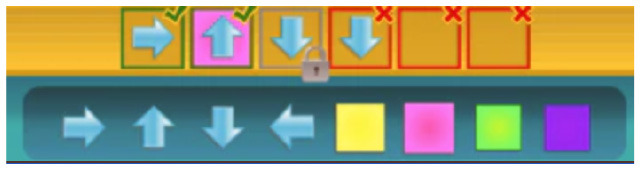
RoboZZle	The background color of the block defines the condition (e.g., “if the tile is red”) and the arrow, the action (e.g., “turn right”).	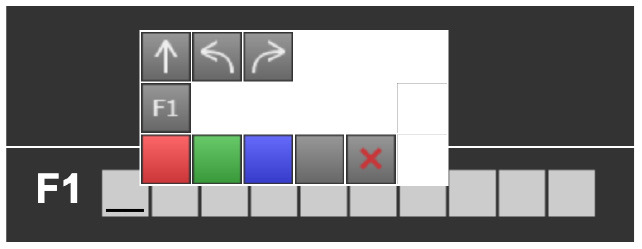
Tynker: Coding for Kids	Predefined condition (e.g., “if snake”) can be combined with an action.	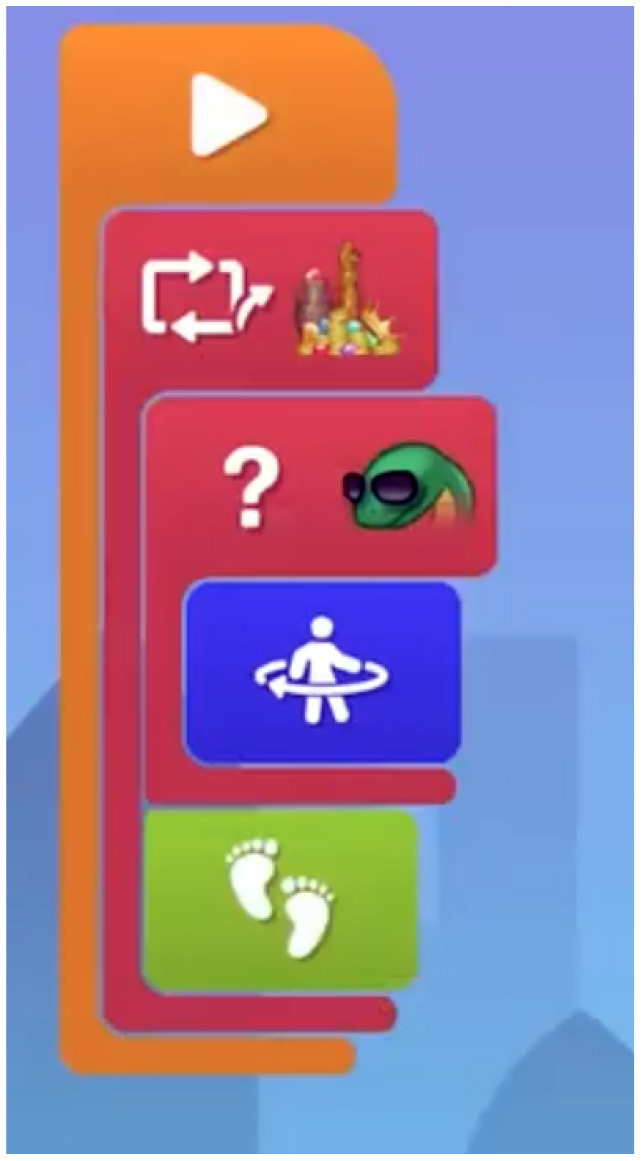
COJI + COJI robot app	Predefined events can be associated with actions, for example, if the head is touched (event that activates procedure 1) - turn and sing (actions defined by the user).	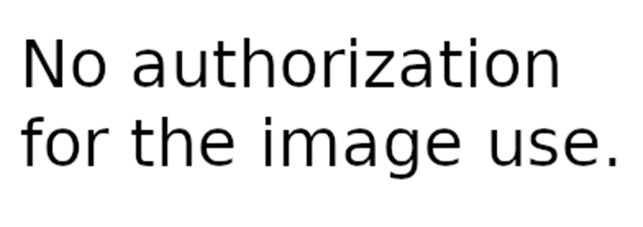
Dash and Dot + Wonder for Dash & Dot Robots	Robot's actions are defined as states and the transition between can be fired based on conditions like “clap heard.”	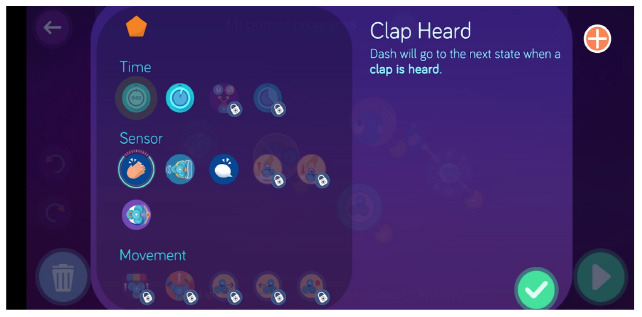
Finch + Finchblox	Blocks attached to the “start when dark” block will be executed when the Finch detects that it is dark.	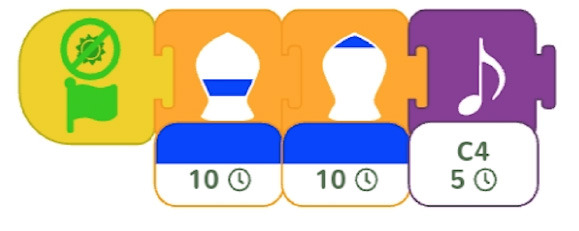
Sphero indi + Sphero Edu Jr	Sphero Edu Jr application allows users to associate a color sensed by the robot with an action involving movement, light, and/or sound.	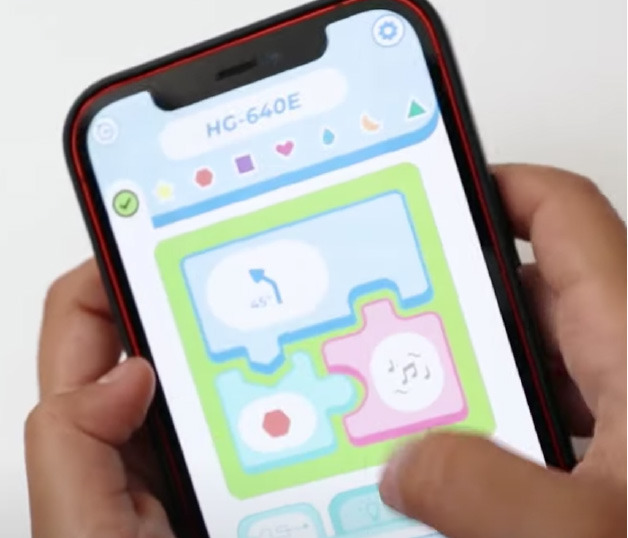
Thymio + Thymio VPL	The user can associate events with actions.	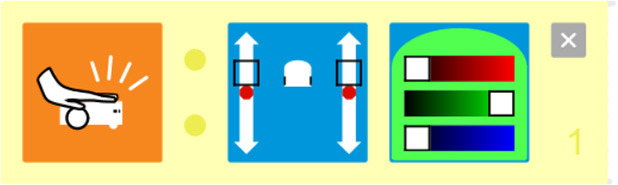
Coding Awbie	Caution Block enables a choice between two sets of sequences based on if there's an obstacle. Image can be found in Getting Started with Osmo Coding Awbie Manual (2022).	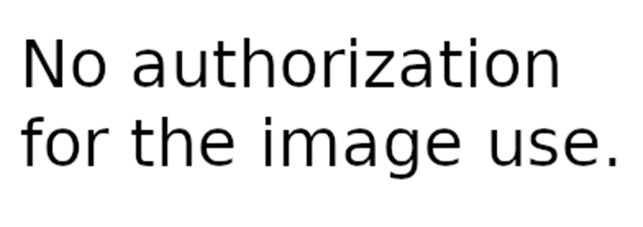
T-maze	“In a program execution, when the avatar reaches one of these squares in the maze, the child must do something with the sensors (e.g., cover a light sensor) to allow the avatar to proceed” Wang et al. (2014).	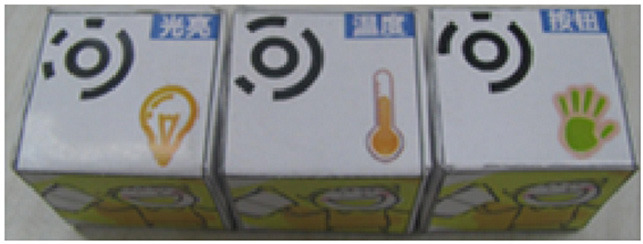

**Table 6:** Tools that allow building conditionals categorized as “Free condition building”.

**Tool**	**Description**	**Reference image**
KIBO	“If” block provides place to add a condition (e.g., far, near, dark, light).	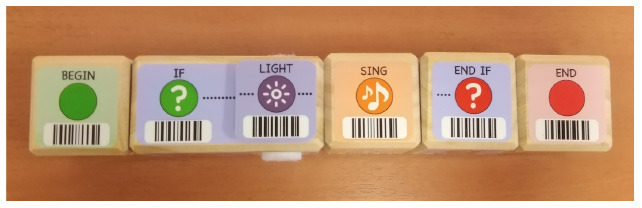
Matatalab Coding Set	“Wait until” can be connected with conditions like: dark, light, obstacle, etc. The block is available in Matatalab Sensor Add-on (2022).	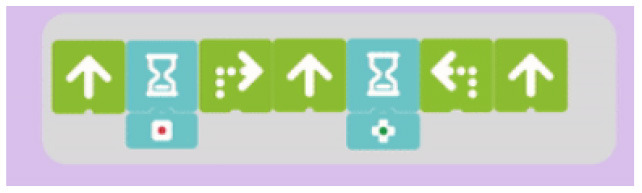
BOTS	“If” block should be associated with variable comparison (e.g., a > 5).	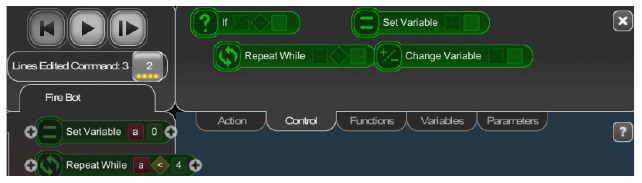
Code.org	The condition in “when tapped” can be modified.	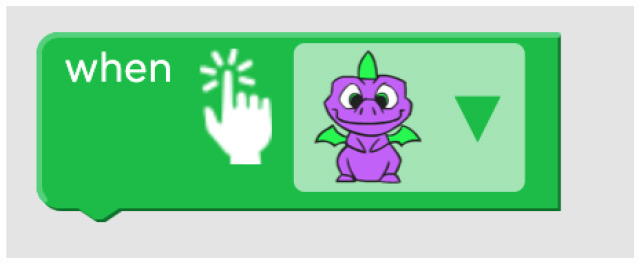
Move the turtle	Condition block evaluates the value of a variable (A > 5).	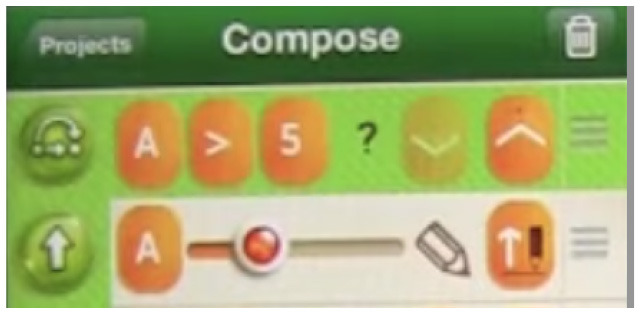
Run Marco!	“If” block can be modified.	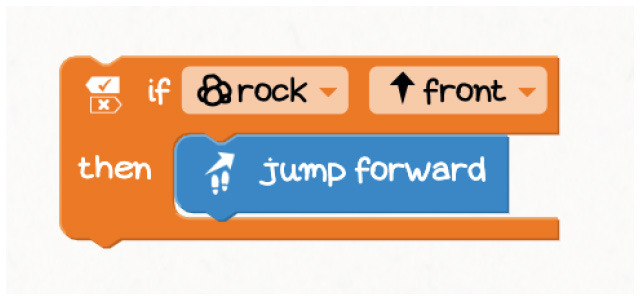
The Foos	The condition is variable and can be changed by the user. A video reference of the implementation can be found on CodeSpark Academy Youtube Channel (2022).	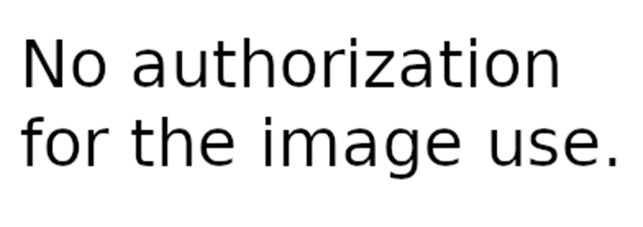
Dash and Dot + Wonder for Dash & Dot Robots	Robot's actions are defined as states and the transition between can be fired based on conditions like “obstacle detection” that can be customized (obstacle seen vs no obstacle, obstacle seen close vs far).	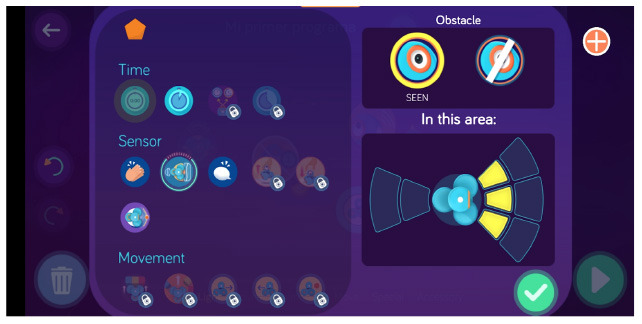
Makeblock Neuron + Neuron (app)	Users can define conditions to establish relations between sensors and actuators.	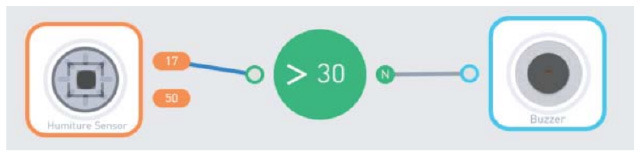
Scottie Go	“If” block should be associated with a specific condition.	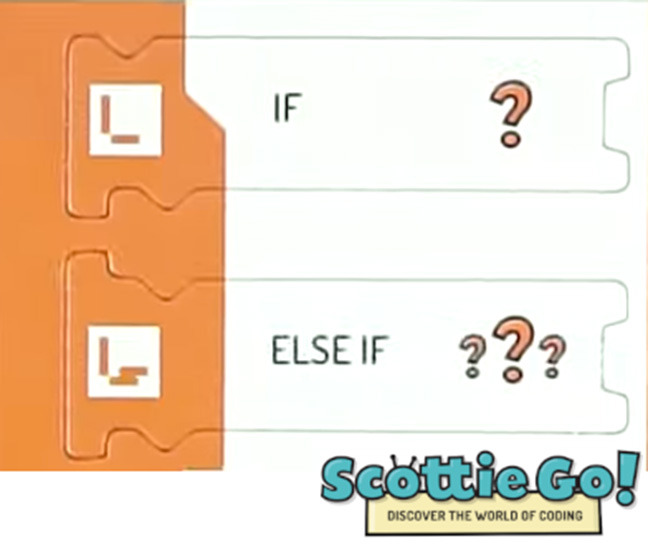
Roberto	“Wait for” can be combined with “tap” event.	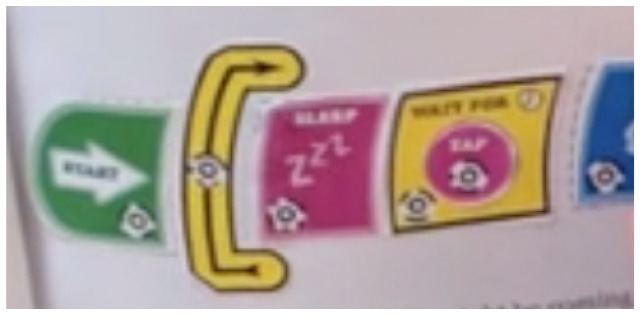

The only tools that enable the definition of conditionals using logical operators (e.g., AND, OR) were Makeblock Neuron and Thymio. Neuron online mode allows users to program behaviors using Neuron App, which supports multiple conditions. In the case of Thymio, the user has to associate events sensed by the robot with its behavior. It is possible to combine the sensing and internal state of the robot (e.g., if Thymio touched AND internal state equal to 1) to program advanced robot responses.

In the case of BOTS, Move the turtle, and Makeblock Neuron + Neuron (app) conditionals are based on numerical variables (e.g., a > 5) which makes them more complex than conditionals with non-numerical conditions (e.g., “if the sensed color is red”), as the children have to understand the concept of variable.

In the case of Coding Awbie, the Caution Block is the only means to introduce conditionals into the code, and is a phased out feature as the block is not included in new kits (Getting Started with Osmo Coding Awbie Manual, 2022).

We also analyzed how the code related to a certain condition interacts with the main program, and identified that they occur within either event-based or procedural programming paradigms. Within event-based programming, we identified the following categories:

- Blocking event: the main program contains a condition that blocks the execution until the condition is fulfilled. For example, KIBO contains a “wait for clap” block that makes the robot wait for a clap before executing commands stored in the following blocks.
- Interruption: the main program is interrupted when a certain event occurs. For example, in the case of Pro-bot the main program is interrupted if the sound sensor is triggered and the procedure associated with this event is executed.
- Parallel execution: It is possible for an event to lead to actions to occur in parallel or in addition to those already occurring. For example, an event in Scratch Jr. could generate a sound while a sprite continues moving on the screen.

Using a procedural programming paradigm, we identified the following category:

- Integrated if: the main program contains conditions expressed using the “if” structure that is evaluated during the program's execution. For example, KIBO allows to incorporate an if-statement into the sequence of commands. If the condition that is evaluated is true, the conditional code is executed and then, the remaining statements.

##### 3.2.2.2. Loops

Another control structure that was relevant for us to analyze was the availability of loops enabling the iteration of commands.

We observed two modalities of implementing the iteration of commands:

- Single command repetition: the tool does not provide the possibility to repeat a sequence of commands, it allows only the repetition of a single action.
- Multiple command repetition: it is possible to repeat multiple commands. In this category we find tools that, due to the design of loop structure, limit the number of pieces that can be repeated (e.g., in Kodable the user is allowed to repeat only two commands) and tools that do not have this restriction.

We also analyzed how the amount of repetitions can be expressed:

- Fixed number of repetitions: the number of repetitions is fixed and cannot be changed by the user.
- Configurable number of repetitions: the amount of repetitions can be defined by the user.
- Infinite loop: it is possible to build infinite loops.

We provide an example for each category in the Table 7.

**Table 7:** Examples of tools for categories developed to classify code iteration.

**Category**	**Description**	**Reference image**
Single command repetition	ScratchJr direction blocks can be modified to make more than one step using single block.	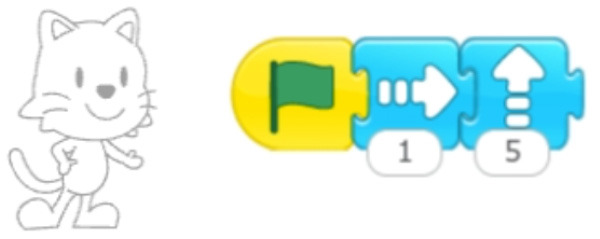
Multiple command repetition	Kodable allows to repeat two commands.	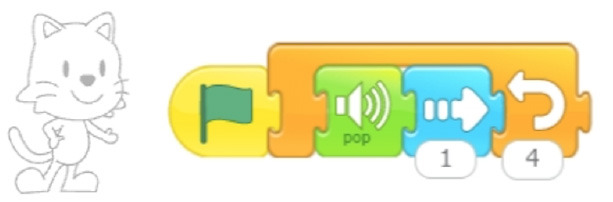
Fixed number of repetitions	Qobo coding card with fixed number of repetitions.	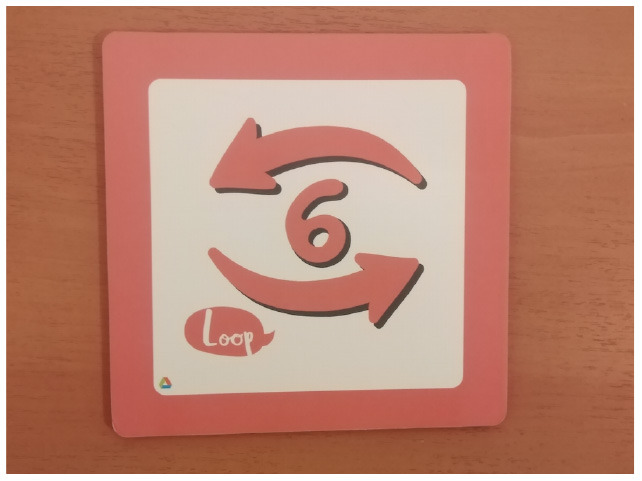
Configurable number of repetitions	Finchblox allows to modify the number of repetitions.	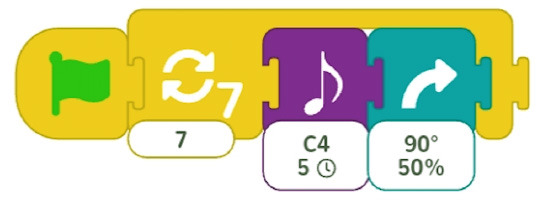
Infinite loop	KIBO allows to associate the repeat block with an infinity symbol.	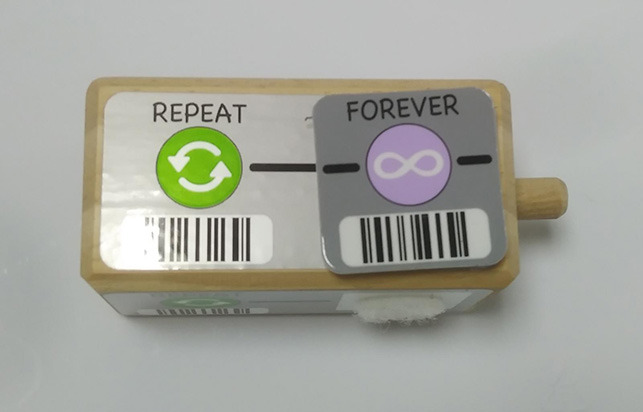

In most cases the amount of repetitions was expressed by associating the number of repetitions with a sequence of statements (similar to a for loop in more advanced programming languages), only BOTS uses exclusively conditions to stop the iteration process (similar to a while loop). KIBO, Finch, Run Marco!, Tynker: Coding for Kids, Scottie Go and VEX 123 offer both types (“repeat X times” and “repeat while”) of repetition statements.

We found many different ways to implement infinite loops: using repeat forever (ScratchJr) or “go to start” command (VEX 123) at the end of the program, elements that contain pieces of code equivalent to “repeat forever” command (Roberto, Code.org), by building circular transitions between states (Dash and Dot), or by calling auxiliary functions (LightbotJr, RoboZZle).

### 3.3. Cost and Availability

Some tools that we analyzed are currently not available for sale: Plobot is a Kickstarter project that finished in Kickstarter (2022), Robotito, BOTS, Roberto, and T-Maze are academic developments, KIWI is KIBO's predecessor and is no longer manufactured, Makeblock Neuron and Puzzlets Starter Pack do not appear in online stores and CHERP is a programming language for KIBO and is not sold separately. All these tools were tagged as “unavailable.”

## 4. SLR of Empirical Evidence (Review 2)

**Figure 4:**
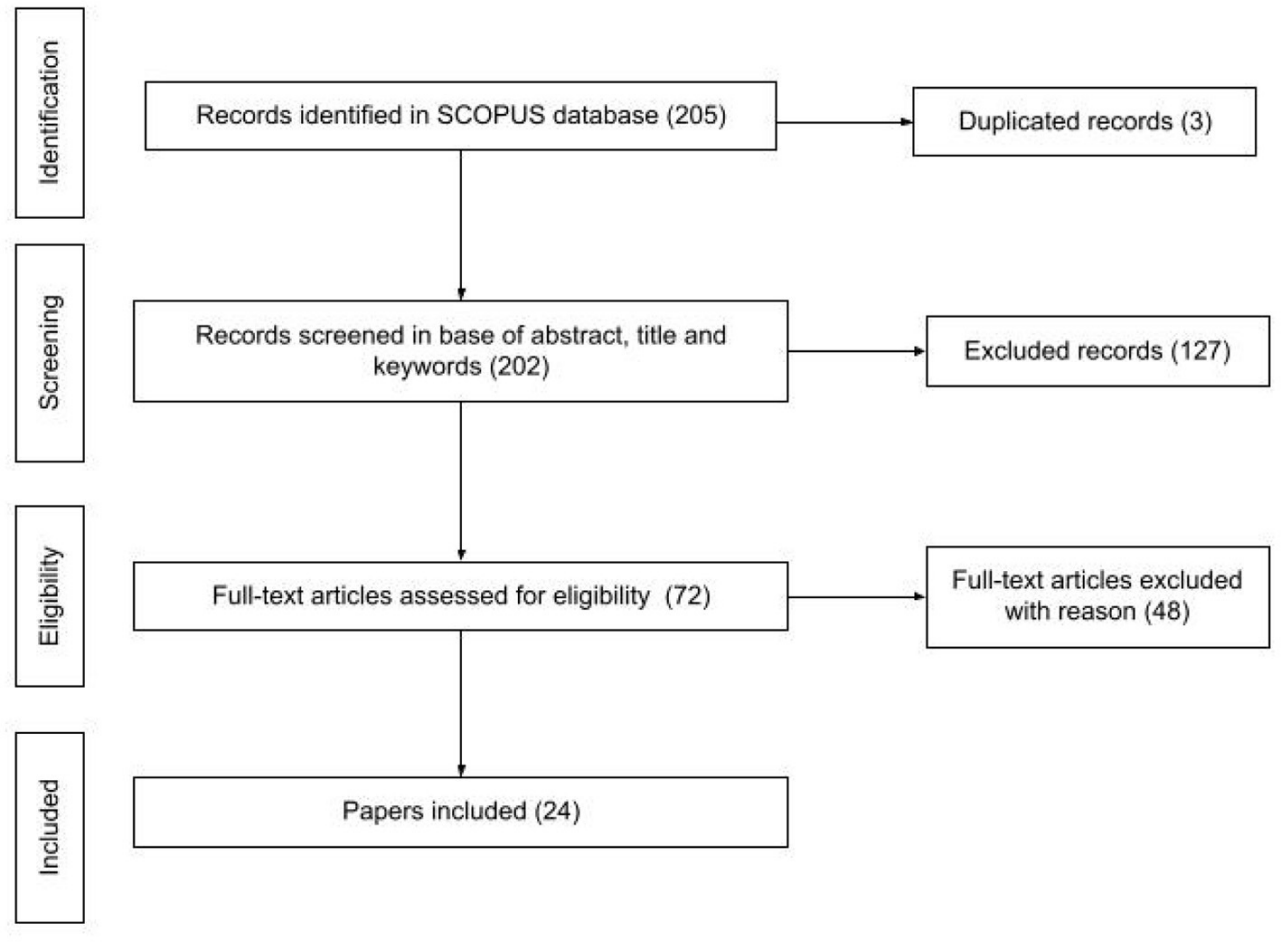
Steps of the selection process of the second SLR. Reported in line with the PRISMA statement (Moher et al., 2009).

We conducted a second SLR (see Figure 4) to identify literature that reports empirical studies with tools that we considered relevant (see Table 3), in which control structures were taught and/or evaluated in order to respond the following research question: What tools have been reported to be successful for teaching control structures?

### 4.1. Methodology

Two reviewers reviewed abstracts and tagged them as “irrelevant” or “relevant.” The last category was used in the cases of publications that meet inclusion criteria or when it was impossible to evaluate the article relevance based on the information available in the abstract. One reviewer reviewed studies that were classified differently among reviewers in the previous step and tried to resolve the doubtful cases. If it was impossible, the articles were considered as “relevant” cases. One reviewer reviewed full-texts of relevant publications and took the final decision about their relevance for this study. We decided not to carry out any quality assessment of the studies as we wanted to provide a broad view of the existing empirical evidence. Two reviewers extracted the data.

#### 4.1.1. Search Strategy

We used an automated search (Kitchenham et al., 2015) in Scopus search engine (Elsevier Scopus, 2022) to identify empirical studies with preschoolers that were developed using tools that we considered relevant (see Table 3). The search term was the following:

TITLE-ABS-KEY ( ( ( {**Tool name**} AND ( preschool OR child OR {early age} OR kindergarten OR {lower education} OR {early years} OR {elementary education} OR {young learner} OR {primary school} OR {primary education} OR k-6 OR k-8 ) ) ) )

It had two keywords: tool name and young learners (and synonyms) and was used to search in title, abstract and keywords.

In some cases we used curly brackets, that limit the search to exact words, ignoring spelling variation or plurals, around the name of the tool ({Tool name}) to avoid false positive results. For example, in the case of “Coffee Platform” when we used Coffee AND Platform instead of Coffee Platform, the results contained irrelevant publications that did not target the robotic platform. In some cases we excluded publications from areas related to medicine, as some tools' names were equal to terms used in medicine and also brought false positive results (as in the case of T-Maze). The search term used and the amount of publications found with each tool can be consulted in appendix.

#### 4.1.2. Study Selection

The inclusion criteria for the studies' selection were the following:

- Articles that report empirical studies with young children using an electronic-based tool that enables activities with control structures.
- Publications that report activities or evaluations focused on control structures.
- Publications focused on children between 3 and 5 years old, including 6 years old, if attending pre-primary school educational level.

Exclusion criteria were:

- Publications that target children older than 6 years.
- Publications that do not report activities or evaluations focused on control structures.
- Off topic articles.
- Articles that describe experiences with users with neurodevelopmental disorders.
- Articles written in a language other than English or Spanish.
- Conference proceedings.

#### 4.1.3. Data Extraction

In the data extraction step we used a spreadsheet to collect information related to the age of participants, number of participants, type of the study, learning outcome, activities aimed at programming conditions, activities that incorporate iterations. Based on the extracted data, two researchers conducted a thematic analysis to summarize study results.

### 4.2. Findings

#### 4.2.1. Scopus Search Result

The Scopus search for all tools was conducted on 13th of October 2021. In many cases the search brought no results. Only 26 tools of 44 unique tools that we identified, counted with Scopus entries (see Appendix). A total of 205 (202 unique) publications were analyzed. Three publications appeared as repeated because the research that they described involved two relevant tools, for example, Pugnali et al.'s research involved KIBO and ScratchJr, so it was found under the search query for KIBO and ScratchJr. We identified 24 unique publications (see Table 8) that met all inclusion criteria. In the screening phase the reviewers identically tagged 152 of 202 unique articles reaching an agreement of 75%.

**Table 8:** 24 relevant publications that we identified in the second SLR.

**References**	**Title**	**Tool name**	**Type of tool**	**Age of participants**	**Number of participants**
Jurado et al. (2020)	Social steam learning at an early age with robotic platforms: A case study in four schools in Spain	KIBO	Physical	4–6	65
Bers (2019)	Coding as another language: a pedagogical approach for teaching computer science in early childhood	KIBO, Scratch Jr	Physical, virtual	4–7	at least 9
Sullivan and Bers (2019)	Investigating the use of robotics to increase girls' interest in engineering during early elementary school	KIBO	Physical	5–7	105
Bers et al. (2019)	Coding as a playground: Promoting positive learning experiences in childhood classrooms	KIBO	Physical	3–5	172
Sullivan and Bers (2018)	Dancing robots: integrating art, music, and robotics in Singapore's early childhood centers	KIBO	Physical	3–6	98
Sullivan et al. (2017)	Imagining, playing, and coding with kibo: Using robotics to foster computational thinking in young children	KIBO	Physical	3–7	322
Pugnali et al. (2017)	THE impact of user interface on young children's computational thinking	KIBO, Scratch Jr	Physical, virtual	4–7	28
Elkin et al. (2016)	Programming with the KIBO Robotics Kit in Preschool Classrooms	KIBO	Physical	3–5	64
Sullivan and Bers (2016b)	Robotics in the early childhood classroom: learning outcomes from an 8–week robotics curriculum in pre–kindergarten through second grade	KIWI	Physical	4–7	60
Sullivan and Bers (2016a)	Girls, boys, and bots: Gender differences in young children's performance on robotics and programming tasks	KIWI, BOTS	Physical, virtual	4–7	45
Strawhacker and Bers (2015)	“I want my robot to look for food”: Comparing Kindergartner's programming comprehension using tangible, graphic, and hybrid user interfaces	CHERP	Hybrid	5–6	35
Kazakoff and Bers (2014)	Put your robot in, put your robot out: Sequencing through programming robots in early childhood	CHERP	Hybrid	4–6	34
Arfé et al. (2020)	The effects of coding on children's planning and inhibition skills	Code.org	Virtual	5–6	179
Çiftci and Bildiren (2020)	The effect of coding courses on the cognitive abilities and problem–solving skills of preschool children	Code.org	Virtual	4–5	28
Pila et al. (2019)	Learning to code via tablet applications: An evaluation of Daisy the Dinosaur and Kodable as learning tools for young children	Kodable, Daisy the Dinosaur	Virtual	4–6	28
Jung et al. (2019)	TurtleTalk: An educational programming game for children with voice user interface	Move the turtle	Virtual	6–9	8
Strawhacker and Bers (2019)	What they learn when they learn coding: investigating cognitive domains and computer programming knowledge in young children	ScratchJr	Virtual	5–8	57
Pinto and Osório (2019)	Learn to program in preschool: Analysis with the participation scale [Aprender a programar en educación infantil: Análisis con la escala de participación]	ScratchJr	Virtual	3–6	71
Strawhacker et al. (2018)	Teaching tools, teachers' rules: exploring the impact of teaching styles on young children's programming knowledge in ScratchJr	ScratchJr	Virtual	5–7	222
Rose et al. (2017)	An exploration of the role of visual programming tools in the development of young children's computational thinking	Game with ScratchJr– and Lightbot–like programming interface	Virtual	6–7	40
Portelance et al. (2016)	Constructing the ScratchJr programming language in the early childhood classroom	ScratchJr	Virtual	5–7	62
Papadakis et al. (2016)	Developing fundamental programming concepts and computational thinking with ScratchJr in preschool education: A case study	ScratchJr	Virtual	4–6	43
Hu et al. (2015)	Strawbies: Explorations in tangible programming	Strawbies	Hybrid	4–10	No info
Wang et al. (2014)	A tangible programming tool for children to cultivate computational thinking	T–maze	Hybrid	5–9	20

The 24 relevant publications reported experiences with 10 different tools that we identified as relevant: ScratchJr (evaluated in 7 articles), KIBO (8), KIWI (2), CHERP (2), Code.org (2), BOTS (1), Kodable (1), Move the turtle (1), Strawbies (1) and T-maze (1). Strawbies is an alternative name for Coding Awbie that was used for the search, as the search term with “Coding Awbie” brought no results. Daisy the Dinosaur was mentioned in a study related to Kodable (Pila et al., 2019), but it targets older children (see Table 2). We also found one case of a custom tool (Rose et al., 2017): a game with both ScratchJr-like and Lightbot style programming interface.

#### 4.2.2. Thematic Analysis

##### 4.2.2.1. KIBO/CHERP/KIWI Articles

The only set of technologies for which control structures have been evaluated multiple times was KIBO/CHERP/KIWI, developed by Marina Bers' group at Tufts University. Of the articles we identified using this technology, five evaluated children's use of control structures while separating the performance of young children from that of older children, or only including children within our inclusion criteria. All these evaluations used the Solve-It assessments, which were developed by the same research group. Through these assessments, in four of the publications, children who fit our inclusion criteria demonstrated proficiency when programming repeat loops (with a given number of repetitions) and “wait for clap” programs, but were not tested on or were unable to be proficient in the use of sensor loops or conditionals (Strawhacker and Bers, 2015; Elkin et al., 2016; Sullivan and Bers, 2016b; Bers et al., 2019). There was one outlying study where children in Kindergarten were able to demonstrate proficiency across all Solve It assessment areas, including repeat loops, sensor loops, “wait for clap” programs, and conditionals (Sullivan and Bers, 2018). Four other evaluations of this tool did not include specific evaluations of control flow (Kazakoff and Bers, 2014; Sullivan et al., 2017; Bers, 2019; Jurado et al., 2020) while two others did not separate children in our age range of interest from older children.

##### 4.2.2.2. Scratch Jr and Others

Most of the other evaluations involved Scratch Jr. (Papadakis et al., 2016; Portelance et al., 2016; Strawhacker et al., 2018; Pinto and Osório, 2019) and did not evaluate children's use or understanding of control structures, even though the tool enables the use of control structures. The same happened with evaluations of other systems (Wang et al., 2014; Hu et al., 2015; Rose et al., 2017; Jung et al., 2019; Pila et al., 2019; Arfé et al., 2020; Çiftci and Bildiren, 2020). The evaluations that did include reports on the use of control structures, without an evaluation, involving Scratch Jr., reported either little use or difficulty with control flow blocks (Pugnali et al., 2017; Strawhacker and Bers, 2019). Another included children in our target age, but also older children without separating their performance (Pugnali et al., 2017). One evaluation of LEGO WeDo found some success with repeat loops, but greater success with CHERP (Strawhacker and Bers, 2015).

##### 4.2.2.3. Bottom Line

Only one study (Sullivan and Bers, 2018) provides evidence of children in Kindergarten mastering conditionals and sensor loops. Multiple studies provide evidence of children in our target age group mastering the use of simple repeat loops (repeat a given # of times) or wait for clap programs. The caveat with all these studies is that they are all from the same research group, use the same system, and the same assessment.

With other tools, except for a study of Lego WeDo which also included CHERP (Strawhacker and Bers, 2015), there are no specific assessments of control flow, other than reports of low use or difficulty with using control flow structures for children in our target age range. In other words, in spite of the great diversity of options for children in our target age range to learn about control flow structures, in our review we found only one technology for which there have been multiple empirical studies to understand whether these children can learn how to use these features.

## 5. Limitations

Although we tried to carry out our study in a systematic way, document all the decisions, and report doubtful cases, the current study still has certain limitations. To complement the tools characteristics related to control structures and cost, we had to appeal to online information. We firstly reviewed official websites and online user manuals, but in some cases the information contained in these sources was not sufficient to answer our research questions. In those cases we reviewed unofficial sources such as youtube videos, blogs and private web pages to complete the missing information. We understand that these are not the most convenient information sources, but we used them if there was no available information through official channels. Another limitation related to our online search is that we reported information that we were able to find, which does not ensure that it is the complete existing information. For example, we reported that the application The Foos allows users to build conditionals of “Free condition building” type based on a youtube video that we found, but we cannot ensure that the tool does not allow building other types of conditionals. There is no free online manual that could provide required information, so to confirm that “Free condition building” is the only type that the tool supports it is necessary to pass all the levels that the game offers, and it was impossible for our team to acquire and personally analyze all the relevant tools. Also, our initial list of tools for young children is limited to the tools reported in scientific publications. It is possible that there are valuable tools that were not mentioned in reviews that we analyzed. We tried to address this issue by adding 3 publications that were not found by SLR and by adding four tools that we found in external sources.

## 6. Discussion

The present study reviewed the state of the art in the teaching of control structures to young children, specifically preliterate children 3 to 6 years of age. While many of the definitions of CT for young children which gather large amounts of consensus amongst academics describe control structures such as conditionals and loops amongst central aspects of CT (Brennan and Resnick, 2012; Grover and Pea, 2013), how this aspect of CT should be developmentally adapted for young children remains unclear. Our findings suggest there is still a large knowledge gap regarding how children acquire early notions about control structures and what the best tools are to introduce children to these concepts. Despite this, these concepts are often included in the interventions targeted at young children and assessed through specific items in the validated CT tests available for young children (Relkin et al., 2020; Zapata-Cáceres et al., 2020).

Our findings demonstrate that there is a wide variety of technological tools which include robots, virtual applications and hybrids, which aim to teach control structures and are targeted to children of these ages. Thus, we infer it is considered relevant that children acquire these concepts early on. Despite this, our findings regarding the reported classroom based research shows that the specifics of how children learn these concepts through the available tools remains unexplored. None of the systematic review articles we identified presented results that were specific to control structures, instead focusing on broader concepts such as CT (Sullivan et al., 2017), programming literacy (Bers, 2019), or engagement (Pinto and Osório, 2019). Given that CT is an umbrella term which encompasses a wide variety of components such as sequencing, using control structures, abstraction, debugging, amongst others (Shute et al., 2017) we must focus on the specifics of each of them in order to have a better sense of the concept as a whole. This is especially relevant for younger children, as the learning curves for each specific skill might differ with age. So far, we found most of the studies focus on several concepts at once but do not further explore learning outcomes for each activity. Thus, the assessments used were more holistic and successful in detecting general learning and engagement outcomes but lacked information on each of the specific tasks and concepts encompassed. An exception to this general approach was the study reported by Kazakoff and Bers (2014) where they focused specifically on sequencing skills, however we did not find any similar study for the learning of control structures, even though our search targeted this term specifically.

Exploring these aspects is also necessary to determine which approaches provide the adequate affordances to enhance learning of each aspect of CT. For example, in our technology overview we observed several approaches to including the use of control structures in tools, such as interrupting events, active wait, or procedural conditions, however there are currently no studies contrasting the strengths and weaknesses of each of these approaches and whether they produce different results in children's understanding of the concepts. As a result, there is only evidence of one tool successfully enabling children to learn some aspects of control structures, mainly due to a lack of studies on the use of other tools by young children that include an assessment of control structure use or understanding.

Moreover, future studies on specific tools should focus on the feasibility of their inclusion in the classrooms in a scalable way. Specifically, our findings regarding the cost of several robots suggest some of them are simply too expensive to be available to all children in a given school or classroom. In addition, some of these tools are more adequately design for individual at-home use, which hinders group based-activities thus elevates the cost of its use even more. Thus, so far the use of robots in education at a large-scale would a entail substantial investment for administrators and policy makers, a problem which could be partially subsided through the design of tools with a group-based focus.

The results of our systematic reviews therefore are encouraging in terms of the wide range of approaches designed for young children to learn about control structures, but also identify a large gap in that we know very little about which of these approaches may work better, or how to structure their use. There is therefore a need for future research to further explore the strengths and weaknesses of the available approaches and understand the feasibility of their use in a variety of contexts (e.g., individual vs. shared, home vs. school).

## 7. Conclusion

The present work demonstrates that there are many diverse tools to support the development of CT in young children. It seems that both academia and industry have interest in designing approaches to enable young children to develop this so-called twenty-first century skill, as we found through our systematic reviews. Although many existing tools allow children to approach advanced programming concepts such as control structures, it is not clear which tools and activities are the most appropriate for teaching them to the youngest programmers. In order to lay the basis for the future research that targets this gap, we provide a systematic overview of existing tools for preliterate children between the ages of 3 and 6. We developed categories that classify the type and complexity of conditionals and iteration structures and used them to categorize each tool. We also provided graphical examples of conditionals that the tools provide.

The analysis of empirical evidence showed that KIBO/CHERP/KIWI is the only tool that consistently demonstrates positive results in teaching control structures to young children. Other tools in our review have not gone through similar evaluations, making it difficult to reach conclusions about their appropriateness for introducing these concepts. The contrast between the diversity of approaches available and the scarcity of evaluations focused on control structures calls for more research, ideally by groups independent of the tools being evaluated, to compare and contrast these approaches in a variety of contexts (e.g., home, preschool).

## Data Availability Statement

The original contributions presented in the study are included in the article/supplementary material, further inquiries can be directed to the corresponding author.

## Author Contributions

EB, AG, JH, and GTe contributed to conception and design of the study. EB organized the first draft of the manuscript and extracted the tools. EB, AG, and JH wrote sections of the manuscript. EB and AG analyzed the articles involved in the first SLR and reviewed tools characteristics. GTe and JH supervised the revision process. GTr and KP analyzed the articles involved in the second SLR. KP and JH extracted the data and conducted the thematic analysis. All authors contributed to manuscript revision, read, and approved the submitted version.

## Funding

This review is a part of a Ph.D. project of the EB that was supported by Comisión Académica de Posgrado (Uruguay).

## Conflict of Interest

The authors declare that the research was conducted in the absence of any commercial or financial relationships that could be construed as a potential conflict of interest.

## Publisher's Note

All claims expressed in this article are solely those of the authors and do not necessarily represent those of their affiliated organizations, or those of the publisher, the editors and the reviewers. Any product that may be evaluated in this article, or claim that may be made by its manufacturer, is not guaranteed or endorsed by the publisher.

## Appendix

**Table A1:** Search term used with each tool to search in SCOPUS.

**Tool name**	**Search term**	**Search results**	**Relevant results**
Bee Bot	TITLE-ABS-KEY ( ( ( Bee bot AND ( preschool OR child OR {early age} OR kindergarten OR {lower education} OR {early years} OR {elementary education} OR {young learner} OR {primary school} OR {primary education} OR k-6 OR k-8 ) ) ) )	23	
Blue Bot	TITLE-ABS-KEY ( ( ( Blue bot AND ( preschool OR child OR {early age} OR kindergarten OR {lower education} OR {early years} OR {elementary education} OR {young learner} OR {primary school} OR {primary education} OR k-6 OR k-8 ) ) ) )	3	
Botley	TITLE-ABS-KEY ( ( ( botley AND ( preschool OR child OR {early age} OR kindergarten OR {lower education} OR {early years} OR {elementary education} OR {young learner} OR {primary school} OR {primary education} OR k-6 OR k-8 ) ) ) )	1	
Code-a-Pillar	TITLE-ABS-KEY ( ( ( {Code-a-Pillar} AND ( preschool OR child OR {early age} OR kindergarten OR {lower education} OR {early years} OR {elementary education} OR {young learner} OR {primary school} OR {primary education} OR k-6 OR k-8 ) ) ) )	0	
Cubetto	TITLE-ABS-KEY ( ( ( {Cubetto} AND ( preschool OR child OR {early age} OR kindergarten OR {lower education} OR {early years} OR {elementary education} OR {young learner} OR {primary school} OR {primary education} OR k-6 OR k-8 ) ) ) )	7	
KIBO	TITLE-ABS-KEY ( ( ( {KIBO} AND ( preschool OR child OR {early age} OR kindergarten OR {lower education} OR {early years} OR {elementary education} OR {young learner} OR {primary school} OR {primary education} OR k-6 OR k-8 ) ) ) )	20	8
KIWI	TITLE-ABS-KEY ( ( ( kiwi AND robot AND ( preschool OR child OR {early age} OR kindergarten OR {lower education} OR {early years} OR {elementary education} OR {young learner} OR {primary school} OR {primary education} OR k-6 OR k-8 ) ) ) )	2	2
KUBO robot	TITLE-ABS-KEY ( ( ( kubo AND robot AND ( preschool OR child OR {early age} OR kindergarten OR {lower education} OR {early years} OR {elementary education} OR {young learner} OR {primary school} OR {primary education} OR k-6 OR k-8 ) ) ) )	0	
Matatalab Coding Set	TITLE-ABS-KEY ( ( ( Matatalab AND robot AND ( preschool OR child OR {early age} OR kindergarten OR {lower education} OR {early years} OR {elementary education} OR {young learner} OR {primary school} OR {primary education} OR k-6 OR k-8 ) ) ) )	0	
mTiny	TITLE-ABS-KEY ( ( ( mtiny AND ( preschool OR child OR {early age} OR kindergarten OR {lower education} OR {early years} OR {elementary education} OR {young learner} OR {primary school} OR {primary education} OR k-6 OR k-8 ) ) ) )	0	
Ozobot Evo	TITLE-ABS-KEY ( ( ( ozobot AND ( preschool OR child OR {early age} OR kindergarten OR {lower education} OR {early years} OR {elementary education} OR {young learner} OR {primary school} OR {primary education} OR k-6 OR k-8 ) ) ) )	6	
Ozobot Bit	considered above		
Plobot	TITLE-ABS-KEY ( ( ( plobot AND ( preschool OR child OR {early age} OR kindergarten OR {lower education} OR {early years} OR {elementary education} OR {young learner} OR {primary school} OR {primary education} OR k-6 OR k-8 ) ) ) )	0	
Pro-bot	TITLE-ABS-KEY ( ( ( pro-bot AND ( preschool OR child OR {early age} OR kindergarten OR {lower education} OR {early years} OR {elementary education} OR {young learner} OR {primary school} OR {primary education} OR k-6 OR k-8 ) ) ) )	0	
Qobo	TITLE-ABS-KEY ( ( ( qobo AND ( preschool OR child OR {early age} OR kindergarten OR {lower education} OR {early years} OR {elementary education} OR {young learner} OR {primary school} OR {primary education} OR k-6 OR k-8 ) ) ) )	0	
Robot Mouse	TITLE-ABS-KEY ( ( ( {Robot Mouse} AND ( preschool OR child OR {early age} OR kindergarten OR {lower education} OR {early years} OR {elementary education} OR {young learner} OR {primary school} OR {primary education} OR k-6 OR k-8 ) ) ) )	0	
Robotito	TITLE-ABS-KEY ( ( ( robotito AND ( preschool OR child OR {early age} OR kindergarten OR {lower education} OR {early years} OR {elementary education} OR {young learner} OR {primary school} OR {primary education} OR k-6 OR k-8 ) ) ) )	2	
Sphero indi	TITLE-ABS-KEY ( ( ( {sphero} AND ( preschool OR child OR {early age} OR kindergarten OR {lower education} OR {early years} OR {elementary education} OR {young learner} OR {primary school} OR {primary education} OR k-6 OR k-8 ) ) ) )	15	
TurtleBot	TITLE-ABS-KEY ( ( ( Turtlebot AND ( preschool OR child OR {early age} OR kindergarten OR {lower education} OR {early years} OR {elementary education} OR {young learner} OR {primary school} OR {primary education} OR k-6 OR k-8 ) ) ) )	4	
VEX 123	TITLE-ABS-KEY ( ( ( {vex 123} AND ( preschool OR child OR {early age} OR kindergarten OR {lower education} OR {early years} OR {elementary education} OR {young learner} OR {primary school} OR {primary education} OR k-6 OR k-8 ) ) ) )	0	
BOTS	TITLE-ABS-KEY ( ( ( {BOTS} AND ( preschool OR child OR {early age} OR kindergarten OR {lower education} OR {early years} OR {elementary education} OR {young learner} OR {primary school} OR {primary education} OR k-6 OR k-8 ) ) ) ) AND ( EXCLUDE ( SUBJAREA , "MEDI" ) )	39	1
Codeable Crafts	TITLE-ABS-KEY ( ( ( {Codeable Crafts} AND ( preschool OR child OR {early age} OR kindergarten OR {lower education} OR {early years} OR {elementary education} OR {young learner} OR {primary school} OR {primary education} OR k-6 OR k-8 ) ) ) )	0	
Code.org	TITLE-ABS-KEY ( ( ( {code.org} AND ( preschool OR child OR {early age} OR kindergarten OR {lower education} OR {early years} OR {elementary education} OR {young learner} OR {primary school} OR {primary education} OR k-6 OR k-8 ) ) ) )	19	2
Kodable	TITLE-ABS-KEY ( ( ( {Kodable} AND ( preschool OR child OR {early age} OR kindergarten OR {lower education} OR {early years} OR {elementary education} OR {young learner} OR {primary school} OR {primary education} OR k-6 OR k-8 ) ) ) )	3	1
LightBotJr	TITLE-ABS-KEY ( ( ( {LightBotJr} AND ( preschool OR child OR {early age} OR kindergarten OR {lower education} OR {early years} OR {elementary education} OR {young learner} OR {primary school} OR {primary education} OR k-6 OR k-8 ) ) ) )	0	
Move the turtle	TITLE-ABS-KEY ( ( ( {move the turtle} AND ( preschool OR child OR {early age} OR kindergarten OR {lower education} OR {early years} OR {elementary education} OR {young learner} OR {primary school} OR {primary education} OR k-6 OR k-8 ) ) ) )	1	1
RoboZZle	TITLE-ABS-KEY ( ( ( {RoboZZle} AND ( preschool OR child OR {early age} OR kindergarten OR {lower education} OR {early years} OR {elementary education} OR {young learner} OR {primary school} OR {primary education} OR k-6 OR k-8 ) ) ) )	0	
Run Marco!	TITLE-ABS-KEY ( ( ( {Run Marco} AND ( preschool OR child OR {early age} OR kindergarten OR {lower education} OR {early years} OR {elementary education} OR {young learner} OR {primary school} OR {primary education} OR k-6 OR k-8 ) ) ) )	2	
ScratchJr	TITLE-ABS-KEY ( ( ( {ScratchJr} AND ( preschool OR child OR {early age} OR kindergarten OR {lower education} OR {early years} OR {elementary education} OR {young learner} OR {primary school} OR {primary education} OR k-6 OR k-8 ) ) ) )	28	7
The Foos	TITLE-ABS-KEY ( ( ( {The Foos} AND ( preschool OR child OR {early age} OR kindergarten OR {lower education} OR {early years} OR {elementary education} OR {young learner} OR {primary school} OR {primary education} OR k-6 OR k-8 ) ) ) )	0	
Tynker: Coding for Kids	TITLE-ABS-KEY ( ( ( {Tynker} AND ( preschool OR child OR {early age} OR kindergarten OR {lower education} OR {early years} OR {elementary education} OR {young learner} OR {primary school} OR {primary education} OR k-6 OR k-8 ) ) ) )	1	
Blue Bot	Repeated tool		
CHERP	TITLE-ABS-KEY ( ( ( cherp AND ( preschool OR child OR {early age} OR kindergarten OR {lower education} OR {early years} OR {elementary education} OR {young learner} OR {primary school} OR {primary education} OR k-6 OR k-8 ) ) ) )	4	2
COJI	TITLE-ABS-KEY ( ( ( {coji} AND ( preschool OR child OR {early age} OR kindergarten OR {lower education} OR {early years} OR {elementary education} OR {young learner} OR {primary school} OR {primary education} OR k-6 OR k-8 ) ) ) )	0	
Dash and/or Dot	TITLE-ABS-KEY ( ( ( {Dash} AND robot AND ( preschool OR child OR {early age} OR kindergarten OR {lower education} OR {early years} OR {elementary education} OR {young learner} OR {primary school} OR {primary education} OR k-6 OR k-8 ) ) ) )	3	
Finch	TITLE-ABS-KEY ( ( ( {finch} AND robot AND ( preschool OR child OR {early age} OR kindergarten OR {lower education} OR {early years} OR {elementary education} OR {young learner} OR {primary school} OR {primary education} OR k-6 OR k-8 ) ) ) )	1	
Sphero indi	Repeated tool		
Thymio	TITLE-ABS-KEY ( ( ( {Thymio} AND ( preschool OR child OR {early age} OR kindergarten OR {lower education} OR {early years} OR {elementary education} OR {young learner} OR {primary school} OR {primary education} OR k-6 OR k-8 ) ) ) )	13	
Tinkerbots	TITLE-ABS-KEY ( ( ( tinkerbots AND ( preschool OR child OR {early age} OR kindergarten OR {lower education} OR {early years} OR {elementary education} OR {young learner} OR {primary school} OR {primary education} OR k-6 OR k-8 ) ) ) )	0	
Makeblock Neuron	TITLE-ABS-KEY ( ( ( makeblock AND neuron AND ( preschool OR child OR {early age} OR kindergarten OR {lower education} OR {early years} OR {elementary education} OR {young learner} OR {primary school} OR {primary education} OR k-6 OR k-8 ) ) ) )	0	
Puzzlets Starter Pack	TITLE-ABS-KEY ( ( ( {Puzzlets} AND ( preschool OR child OR {early age} OR kindergarten OR {lower education} OR {early years} OR {elementary education} OR {young learner} OR {primary school} OR {primary education} OR k-6 OR k-8 ) ) ) )	0	
Roberto	TITLE-ABS-KEY ( ( ( {Roberto} AND ( preschool OR child OR {early age} OR kindergarten OR {lower education} OR {early years} OR {elementary education} OR {young learner} OR {primary school} OR {primary education} OR k-6 OR k-8 ) ) ) ) AND ( EXCLUDE ( SUBJAREA,"MEDI" ) OR EXCLUDE ( SUBJAREA,"NURS" ) OR EXCLUDE ( SUBJAREA,"NEUR" ) OR EXCLUDE ( SUBJAREA,"PHAR" ) OR EXCLUDE ( SUBJAREA,"IMMU" ) OR EXCLUDE ( SUBJAREA,"BIOC" ) ) AND ( EXCLUDE ( SUBJAREA,"ARTS" ) OR EXCLUDE ( SUBJAREA,"SOCI" ) )	5	
Scottie Go	TITLE-ABS-KEY ( ( ( {Scottie Go} AND ( preschool OR child OR {early age} OR kindergarten OR {lower education} OR {early years} OR {elementary education} OR {young learner} OR {primary school} OR {primary education} OR k-6 OR k-8 ) ) ) )	0	
Coding Awbie	TITLE-ABS-KEY ( ( ( {strawbies} AND ( preschool OR child OR {early age} OR kindergarten OR {lower education} OR {early years} OR {elementary education} OR {young learner} OR {primary school} OR {primary education} OR k-6 OR k-8 ) ) ) )	1	1
Tabletop puzzle block system	TITLE-ABS-KEY ( ( ( {Tabletop puzzle} AND ( preschool OR child OR {early age} OR kindergarten OR {lower education} OR {early years} OR {elementary education} OR {young learner} OR {primary school} OR {primary education} OR k-6 OR k-8 ) ) ) )	1	
T-Maze	TITLE-ABS-KEY ( ( ( {t-maze} AND ( preschool OR child OR {early age} OR kindergarten OR {lower education} OR {early years} OR {elementary education} OR {young learner} OR {primary school} OR {primary education} OR k-6 OR k-8 ) ) ) ) AND ( EXCLUDE ( SUBJAREA , "BIOC" ) OR EXCLUDE ( SUBJAREA , "MEDI" ) OR EXCLUDE ( SUBJAREA , "PHAR" ) ) AND ( EXCLUDE ( SUBJAREA , "NEUR" ) )	1	1
